# Coronavirus disease 2019 (COVID-19) research agenda for healthcare epidemiology

**DOI:** 10.1017/ice.2021.25

**Published:** 2021-01-25

**Authors:** Lona Mody, Ibukunoluwa C. Akinboyo, Hilary M. Babcock, Werner E. Bischoff, Vincent Chi-Chung Cheng, Kathleen Chiotos, Kimberly C. Claeys, K. C. Coffey, Daniel J. Diekema, Curtis J. Donskey, Katherine D. Ellingson, Heather M. Gilmartin, Shruti K. Gohil, Anthony D. Harris, Sara C. Keller, Eili Y. Klein, Sarah L. Krein, Jennie H Kwon, Adam S. Lauring, Daniel J. Livorsi, Eric T. Lofgren, Katreena Merrill, Aaron M. Milstone, Elizabeth A. Monsees, Daniel J. Morgan, Luci P. Perri, Christopher D. Pfeiffer, Clare Rock, Sanjay Saint, Emily Sickbert-Bennett, Felicia Skelton, Katie J. Suda, Thomas R. Talbot, Valerie M. Vaughn, David J. Weber, Timothy L. Wiemken, Mohamed H. Yassin, Matthew J. Ziegler, Deverick J. Anderson

**Affiliations:** 1 Division of Geriatric and Palliative Medicine, Department of Internal Medicine, University of Michigan, Ann Arbor, Michigan, United States; 2 Geriatrics Research Education and Clinical Center, Veterans’ Affairs Ann Arbor Healthcare System, Ann Arbor, Michigan, United States; 3 Division of Infectious Diseases, Department of Pediatrics, Duke University School of Medicine, Durham, North Carolina, United States; 4 Washington University School of Medicine, St. Louis, Missouri, United States; 5 Wake Forest School of Medicine, Winston Salem, North Carolina, United States; 6 Department of Microbiology, Queen Mary Hospital, Hong Kong Special Administrative Region, China; 7 Infection Control Team, Queen Mary Hospital, Hong Kong West Cluster, Hong Kong Special Administrative Region, China; 8 Division of Critical Care Medicine, Children’s Hospital of Philadelphia, Philadelphia, Pennsylvania, United States; 9 University of Maryland School of Pharmacy, Baltimore, Maryland, United States; 10 University of Maryland School of Medicine, Baltimore, Maryland, United States; 11 Carver College of Medicine, University of Iowa, Iowa City, Iowa, United States; 12 Infectious Diseases Section, Louis Stokes Cleveland Veterans’ Affairs Medical Center, Cleveland, Ohio, United States; 13 Case Western Reserve University School of Medicine, Cleveland, Ohio, United States; 14 Department of Epidemiology and Biostatistics, College of Public Health, University of Arizona, Tucson, Arizona, United States; 15 Veterans’ Affairs Eastern Colorado Healthcare System, Aurora, Colorado, United States; 16 Colorado School of Public Health, University of Colorado, Aurora, Colorado, United States; 17 Division of Infectious Diseases, University of California Irvine School of Medicine, Irvine, California, United States; 18 Epidemiology and Infection Prevention, UC Irvine Health, Irvine, California, United States; 19 Division of Infectious Diseases, John Hopkins University School of Medicine, Baltimore, Maryland, United States; 20 Department of Emergency Medicine, Johns Hopkins University, Baltimore, Maryland, Unites States; 21 Veterans’ Affairs Ann Arbor Center for Clinical Management Research, Ann Arbor, Michigan, United States; 22 Department of Internal Medicine, University of Michigan, Ann Arbor, Michigan, United States; 23 Division of Infectious Diseases, Department of Internal Medicine, University of Michigan, Ann Arbor, Michigan, United States; 24 Iowa City Veterans’ Affairs Health Care System, Iowa City, Iowa, United States; 25 Paul G. Allen School for Global Animal Health, Washington State University, Pullman, Washington, United States; 26 Brigham Young University, Provo, Utah, United States; 27 Division of Pediatric Infectious Diseases, Johns Hopkins University School of Medicine, Baltimore, Maryland, United States; 28 Children’s Mercy Kansas City, Kansas City, Missouri, United States; 29 University of Missouri–Kansas City School of Medicine, Kansas City, Missouri, United States; 30 Veterans’ Affairs Maryland Healthcare System, Baltimore, Maryland, United States; 31 Infection Control Results, Wingate, North Carolina, United States; 32 Veterans’ Affairs Portland Health Care System, Portland, Oregon, United States; 33 Oregon Health & Science University, Portland, Oregon, United States; 34 Veterans’ Affairs Ann Arbor Healthcare System, Ann Arbor, Michigan, United States; 35 Department of Infection Prevention, University of North Carolina Medical Center, Chapel Hill, North Carolina, United States; 36 Michael E. DeBakey Veterans’ Affairs Medical Center, Houston, Texas, United States; 37 H. Ben Taub Department of Physical Medicine & Rehabilitation, Baylor College of Medicine, Houston, Texas, United States; 38 Center for Health Equity Research and Promotion, Veterans’ Affairs Pittsburgh Healthcare System, Pittsburgh, Pennsylvania, United States; 39 Division of General Internal Medicine, University of Pittsburgh School of Medicine, Pittsburgh, Pennsylvania, United States; 40 Vanderbilt University School of Medicine, Nashville, Tennessee, United States; 41 Division of General Internal Medicine, Department of Internal Medicine, University of Utah School of Medicine, Salt Lake City, Utah, United States; 42 University of North Carolina at Chapel Hill, Chapel Hill, North Carolina, United States; 43 Division of Infectious Diseases, Allergy, and Immunology, Department of Medicine, Saint Louis University School of Medicine, St Louis, Missouri, United States; 44 School of Medicine, University of Pittsburgh, Pittsburgh, Pennsylvania, United States; 45 Infectious Diseases and Microbiology, Graduate School of Public Health, University of Pittsburgh, University of Pittsburgh, Pittsburgh, Pennsylvania, United States; 46 Infectious Diseases Division, University of Pennsylvania, Philadelphia, Pennsylvania; 47 Duke Center for Antimicrobial Stewardship and Infection Prevention, Duke University School of Medicine, Durham, North Carolina, United States

## Abstract

This SHEA white paper identifies knowledge gaps and challenges in healthcare epidemiology research related to coronavirus disease 2019 (COVID-19) with a focus on core principles of healthcare epidemiology. These gaps, revealed during the worst phases of the COVID-19 pandemic, are described in 10 sections: epidemiology, outbreak investigation, surveillance, isolation precaution practices, personal protective equipment (PPE), environmental contamination and disinfection, drug and supply shortages, antimicrobial stewardship, healthcare personnel (HCP) occupational safety, and return to work policies. Each section highlights three critical healthcare epidemiology research questions with detailed description provided in supplementary materials. This research agenda calls for translational studies from laboratory-based basic science research to well-designed, large-scale studies and health outcomes research. Research gaps and challenges related to nursing homes and social disparities are included. Collaborations across various disciplines, expertise and across diverse geographic locations will be critical.

The emergence and rapid worldwide spread of severe acute respiratory syndrome coronavirus 2 (SARS-CoV-2) has led to substantial social and economic disruption and loss of life. Throughout the pandemic, healthcare providers, hospitals, and health systems have worked tirelessly to provide safe care for patients while simultaneously ensuring safety for frontline providers. Early efforts to prevent transmission relied on prepandemic evidence and rapidly emerging novel data. Through the first 9 months of the pandemic, >60,000 articles were published on SARS-CoV-2 and coronavirus disease 2019 (COVID-19), not including the now ubiquitous pre-prints.^1^ As a result, the scientific community already has learned a great deal about COVID-19, leading to evolving guidelines for treatment, testing, and prevention.^2–5^ Despite considerable progress, the community still has much to learn.

As the adage goes, “The more you know, the more you realize how much you don’t know.” This SHEA white paper identifies remaining knowledge gaps and challenges in healthcare epidemiology research related to COVID-19. These gaps are described in 10 sections : epidemiology, outbreak investigation, surveillance, isolation precaution practices, personal protective equipment (PPE), environmental contamination and disinfection, drug and supply shortages, antimicrobial stewardship, healthcare personnel (HCP) occupational safety, and return to work policies (Table 1; Supplementary Tables 1–4 online). Each section highlights 3 critical healthcare epidemiology research questions, while many other important research questions are provided in supplementary tables, including a table highlighting the subset of questions particularly relevant to children (Supplementary Table 5 online). Research gaps and challenges related to nursing homes and social disparities are included.

**Table 1.** COVID-19 Healthcare Epidemiology Research Priorities

Section	Research Priority Areas	Detailed Research Questions
**Adult populations**		
Epidemiology	Heterogeneity in transmission dynamics	Supplementary Table 1
	Role of asymptomatic/presymptomatic transmission	
	Risk factors for severe disease outcomes	
Outbreak investigation	Outbreak cessation	
	Personnel and skill set	
	Resources and tools	
Surveillance	Detection strategies	
	Population-level transmission risks	
	Population surveillance	
Isolation precaution practices	Initiation of precautions	Supplementary Table 2
	Management	
	Discontinuation	
Personal protective equipment (PPE)	Determination of appropriate PPE	
	Strategies to improve PPE	
	Impact on other viruses	
Environmental disinfection	Risks related to environmental contamination	
	Strategies for evaluating environmental contamination	
	Disinfection strategies	
Shortages	Impact of shortages	Supplementary Table 3
	Dissemination of best practices	
	Clinical consequences	
Antimicrobial stewardship	Healthcare utilization	
	Coinfection	
	Effective stewardship strategies	
Occupational safety	HCP exposure	Supplementary Table 4
	HCP mental health	
	Social/Organizational barriers and facilitators	
Return to work policies	Risk in returning HCP	
	Optimal criteria for return to work	
	Sociocultural impact	
**Pediatric populations**	Risk of disease transmission	Supplementary Table 5
	Estimate and mitigate transmission in schools	
	Long-term sequelae of symptomatic/asymptomatic disease Risk factors for and long-term sequelae of MIS-C?	
	Develop of effective screening algorithms to initiate isolation	
	Significance of fecal shedding and environmental contamination	
	Impact of COVID-19 on antibiotic prescribing?	

Note. HCP, healthcare personnel; PPE, personal protective equipment; MIS-C, multisystem inflammatory syndrome in children (MIS-C).

## 1. Epidemiology

Understanding the epidemiology of SARS-CoV-2 is critical to minimizing the burden of the COVID-19 pandemic in healthcare settings. Epidemiologic research on individual as well as population-level transmission dynamics, risk factors for virus acquisition, and predictors of severe disease outcomes can inform healthcare capacity planning, clinical care, and infection prevention practices within healthcare settings. Three research domains identified below (Supplementary Table 1 online) represent priority areas with unanswered questions in the epidemiology of disease relevant to healthcare and infection prevention:

*Priority area 1:* Understand heterogeneity in epidemiology and transmission dynamics of SARS-CoV-2.
*Priority area 2:* Define characteristics and impact of asymptomatic/pre-symptomatic patients infected with SARS-CoV-2.
*Priority area 3:* Characterize risk factors that lead to severe disease outcomes including age, sex, and race, with special emphasis on health disparities, socio-economic status, and comorbidities.

The heterogeneity in COVID-19 transmission dynamics is typified by both strain differences and “superspreading events” in which a small number of individuals account for a large fraction of transmission. Recent reports of variant strains being associated with an increased facility for spread as well as higher viral burdens in infected individuals require further explanation. Identifying the causes of superspreading in healthcare settings is key, especially in various settings that house high-risk populations such as in nursing homes and other long-term care facilities.^6–8^ Also critical is an enhanced understanding of viral transmission patterns through the air (in more depth than simply droplet versus airborne transmission) that can support evidence-based PPE, physical distancing, and ventilation policies, which currently vary across healthcare settings.^6^ With continued shortages in PPE, identifying the relative risk of occupational versus community exposures in HCP is essential to identify failures in occupational safety and implement comprehensive interventions to safeguard HCP.

One unique epidemiologic feature of COVID-19 is the sheer number of asymptomatic or presymptomatic cases reported, which has ranged from 1% to >50% and has resulted in widespread increases in SARS-CoV-2 testing.^6,9–15^ Several studies have documented high viral loads in asymptomatic individuals, which suggests that they could be significant contributors to transmission and that symptom screening alone cannot contain transmission.^9,16^ This issue highlights the need for studies in children, who have lower rates of illness and hospitalization and gather in school and daycare settings (Supplementary Table 5 online).^17^ An understanding of the role of asymptomatic or presymptomatic individuals in transmission will influence societal considerations regarding opening schools, resuming economic activities such as opening gyms, and allowing social events such as having small and large gatherings.

Finally, studying the epidemiology of severe and post-acute disease can inform patient and HCP safety protocols, clinical practice guidelines, vaccine recommendations, and concurrent management of other chronic conditions. To date, the burden of SARS-CoV-2 infection has had a disproportionate impact on racial and ethnic minority communities, frontline workers, and individuals with underlying conditions, such as diabetes, hypertension, obesity, and heart, lung, or kidney disease.^7,18–21^ Prioritizing research on the underlying societal and biological risk factors and optimal prevention and treatment for these high-risk groups is important. Furthermore, the extent and burden of long-term cardio-metabolic, respiratory, neurological, and psychological sequelae, including among asymptomatic individuals or those with mild disease, requires further study.^8,22,23^

## 2. Outbreak investigation

COVID-19 poses a unique challenge in outbreak investigation stemming from its novelty coupled with the rapid worldwide spread into all sectors of society, including into diverse healthcare settings in which patients, staff, and visitors can be the source of infection. As a result of asymptomatic transmission of uncertain route, COVID-19 requires a broad new approach to outbreak mitigation within healthcare settings, building upon the traditional public health methodologies. Many important potential avenues of research have opened in pursuit of this aim.^24^ Three research domains identified below (Supplementary Table 1 online) represent priority areas with unanswered questions about optimal COVID-19 outbreak investigations relevant to hospitals, nursing homes, and rehabilitation hospitals:

*Priority area 1:* Identify critical interventions required to stop COVID-19 outbreaks.
*Priority area 2:* Determine optimal personnel, expertise, and training required to conduct rapid SARS-CoV-2 and other outbreak investigations.
*Priority area 3:* Identify optimal resources and technology (reporting tools, software and hardware) to support outbreak investigations.

Several studies have highlighted the need for adequate resources, infrastructure, and personnel with expertise and leadership support to conduct timely, evidence-based infection prevention activities, including outbreak investigations.^25–28^ HCP that work in overtaxed health care systems, faced with a rapidly spreading outbreak, as well as confusing and changing guidance, are at an elevated risk for burnout and moral distress.^29^ These challenges to conducting rapid and effective outbreak investigations are further amplified in nursing homes and small to mid-sized hospitals.^30^ Compared to larger hospitals, smaller hospitals face unique challenges, including infection preventionists (IPs) with other non–infection-related responsibilities, lack of specific IP training, lack of data synthesis and reporting tools, and high personnel turnover.^28,31–33^ For example, rapid reporting systems can provide benchmarks to improve early outbreak detection in hospitals, nursing homes, and other healthcare settings leading to early interventions to curtail the outbreak.^34^ Technical knowhow and expertise in conducting outbreak investigations are important to identify key characteristics of the outbreak, including: populations being most affected; unique presentations that could vary by age, gender, race, comorbidities, or frailty; and patterns of transmission. Such expertise should also provide institutions with rapid, simple, systemic and culturally appropriate interventions.

## 3. Surveillance strategies

Robust surveillance of COVID-19 is critical to designing effective strategies for timely identification of COVID-19, limiting the spread of disease, and informing public health priorities and responses. Three research domains identified below (Supplementary Table 1 online) represent priority areas with unanswered questions in surveillance strategies relevant to healthcare and infection prevention:

*Priority area 1:* Determine optimal and rapid surveillance strategies to accurately define the scale and depth of COVID-19 and its impact on populations, communities, and individuals.
*Priority area 2:* Determine and evaluate high-yield, cost-effective, and efficient testing-based population surveillance strategies.
*Priority area 3:* Identify highest risk populations for targeted interventions based on their age, gender, race, comorbidities, settings, and community spread.

Reverse transcription polymerase chain reaction (RT-PCR) tests for SARS-CoV-2 can remain positive up to 3 months and do not directly translate to transmissibility. Viable virus has often not been found beyond 10 days in immunocompetent hosts, barring some instances.^35–40^ As a result, use of RT-PCR results for surveillance would overestimate COVID-19 incidence and prevalence, leading to misclassification of community-level burden. Large-scale longitudinal surveillance studies are needed to evaluate duration of test positivity (ie, RT-PCR, antigen, and serology) and risk for COVID-19 reinfection, with subgroup evaluation by symptoms (eg, asymptomatic, mild-to-moderate symptoms, and hospitalized patients).^39,40^

Although manufacturers report high sensitivity and specificity against assay controls, clinical sensitivity and specificity for COVID-19 infection is relatively unknown. In some instances, sensitivity has been reported to be as low as 70%, depending on the quality of the specimen obtained and the time at which the sample is taken during a patient’s illness.^41,42^ Studies are needed to evaluate the clinical performance characteristics of COVID-19 testing tools against the sensitivity and specificity of full-symptom screening, including early indicators of infection. Results from these studies will inform optimal sentinel surveillance strategies for large populations through en masse testing, such as pooled saliva sample testing or sewer line sampling.

Large-scale surveillance data within a wide variety of community and work settings and activities can lead to identification of groups and locations associated with high risk for transmission, leading to improved strategies for prevention and PPE use. Specific attention is needed within healthcare settings including nursing homes; assisted living facilities; group homes; factories and food processing plants; jails and prisons; and places of education such as schools, colleges, and universities. Supplementary Table 5 (online) highlights additional considerations relevant to pediatric surveillance, including surveillance for the multisystem inflammatory syndrome in children (MIS-C). Exposure risks may be further defined through novel surveillance tools (eg, personal exposure monitors and tracking apps).

## 4. Isolation precaution practices

Standard and transmission-based precaution practices are cornerstones of preventing transmission of infectious pathogens and ensuring HCP and patient safety across all healthcare settings.^43^ The US Centers for Disease Control and Prevention (CDC) developed and updated interim infection prevention and control recommendations regarding the use of transmission-based isolation precautions when caring for patients with suspected or confirmed SARS-CoV-2 infection in healthcare facilities.^44^ This guidance focuses on HCP and patient screening, testing protocols, patient placement and management practices, use of PPE, and family/visitor interactions.

However, as the COVID-19 pandemic continues to unfold, so does the need for a more rigorous evidence base to inform isolation practices and to assist healthcare facilities with effectively implementing public health guidance. Three research domains identified here (Supplementary Table 2 online) represent priority areas with unanswered questions in isolation precautions relevant to healthcare and infection prevention:

*Priority area 1:* Determine when and how to initiate transmission-based isolation precautions for COVID-19.
*Priority area 2:* Determine how to optimize management and care delivery while isolation precautions are in place.
*Priority area 3:* Determine when to discontinue COVID-19 isolation precautions and reinstitute isolation in cases of possible re-infection.

COVID-19 has a wide variety of clinical presentations ranging from asymptomatic to severely ill.^45^ Healthcare facilities use various criteria based on individual signs and symptoms to determine when to test individuals for COVID-19 and initiate isolation precautions while awaiting results, and they use various testing protocols to detect asymptomatic and presymptomatic individuals. Although these are critical strategies for stopping COVID-19 transmission, questions remain about the effectiveness of various screening and testing protocols to initiate isolation precautions practices and reduce transmission risk.

Once in isolation, use of PPE (ie, gloves, gown, mask, N95 respirator or power air-purifying respirator [PAPR], eye protection) for known or suspected COVID-19 patients can pose challenges to the delivery of care and can potentially delay recognition of other healthcare-associated conditions.^46^ Furthermore, COVID-19 isolation can be problematic for hospitalized patients and nursing home residents due to the use of equipment that can inhibit visual and auditory cues and visitor restrictions resulting in less family contact and support. The inability to connect with family members is one of the most distressing consequences of COVID-19 isolation, with a potential for long-lasting psychological consequences in survivors.^47,48^ Research to better understand, identify, and test approaches to mitigate the psychological, physical, and care delivery challenges related to COVID-19 isolation precaution practices, including the benefits and unintended consequences of family and visitor policies and restrictions, are needed.^46–49^

Recommendations for discontinuing isolation have been based on symptoms, test results, and time from positive test.^37,50,51^ Discontinuation of isolation precautions allows individuals to engage in normal and/or recovery-focused activities. To do so safely, however, discontinuation policies must also take into account the risk of secondary transmission. In other words, balancing risk of transmission events if isolation is discontinued too early against the risks of staying in isolation too long. Thus, research on when and how to safely discontinue isolation that balances public health and patient priorities is needed. Improved understanding of how continued positive test results correlate with transmission will also help guide isolation discontinuation policies.^52^

These research questions and resultant policy implications impact HCP directly. Early studies suggest that interventions, such as a triage committee and team decision making, may decrease the perception of personal culpability for untoward patient outcomes.^29^ Thus, meaningfully enhancing HCP engagement in developing research questions and in decision-making processes are critical to reducing moral distress and burnout.

## 5. Personal protective equipment (PPE)

General recommendations for HCP use of PPE are available from the CDC; expert groups have provided additional recommendations for use of PPE in crisis scenarios.^5,44^ Both CDC and expert guidance has been largely based on limited data extrapolated from other viral infections (eg, influenza, SARS-CoV-1) and/or studies with significant biases limiting generalizability. Research domains identified below (Supplementary Table 2 online) represent priority areas with unanswered questions concerning PPE use in healthcare settings:

*Priority area 1:* What is the appropriate level of universal PPE for current pandemic and afterward?
*Priority area 2:* What are the sociobehavioral, adaptive, and contextual factors required to improve appropriate PPE use?
*Priority area 3:* Are PPE interventions made during the COVID-19 pandemic likely to be effective against other commonly circulating respiratory viruses?

COVID-19 is generally thought to spread primarily through respiratory droplets; thus, the current role of PPE is aimed at decreasing droplet transmission. Masks are used as the cornerstone of source control (symptomatic or asymptomatic person with COVID-19).^53^ However, it is possible that COVID-19 transmission can occur through the eyes, either by direct droplet inoculation or via autoinoculation. In this setting, eye protection (face shields or goggles) has also been recommended and may play a key role in infection prevention. In a 2014 study that utilized a cough simulator and breathing worker simulator to model droplet transmission, face shields prevented exposure to droplets; however, masks were not used.^54^ A meta-analysis indicated that physical distancing, masks, and eye protection decreased the odds of COVID-19 transmission; however, the relative risk reduction of eye protection plus a face mask for COVID-19 has not been well described.^55^ Additionally, the benefit of face shields alone in source control of an asymptomatic or presymptomatic patient is not known.

Research on compliance with PPE guidance in prior outbreaks has focused on methods of delivering training.^56^ In the Ebola virus disease outbreak, a human-factors engineering approach to training and ensuring appropriate donning and doffing decreased ambiguity, explored failure modes, and enhanced teamwork to improve compliance with PPE guidance.^57^ Thus, other behavioral, adaptive, cultural, systemic or other factors may play a role in adherence to best-practice PPE use. Studies that utilize methods from healthcare epidemiology, infection prevention, human factors engineering, and medical sociology are needed to identify and mitigate (or enhance) sociobehavioral, adaptive, contextual, and human factors that impact appropriate PPE use. In the current pandemic, where mask-wearing has become politicized, understanding these factors may be even more essential. Understanding how to improve appropriate PPE use, through understanding the socio-behavioral, adaptive, and contextual reasons for not following appropriate PPE guidance, as well as human factors associated with appropriate PPE use, could improve PPE adherence among HCP. Finally, PPE use has been complicated by shortages, which resulted in institutions requiring healthcare providers to reuse single-use items such as N-95 masks. The shortage of essential supplies can increase anxiety and fear in those who need them; however, the impact of PPE shortages on future use of PPE is a consideration that requires further investigation.^58–60^

## 6. Environmental contamination and disinfection

Surface contamination with SARS-CoV-2 has been frequently described,^61–74^ but the role of environmental contamination with SARS-CoV-2 in transmission in healthcare settings such as hospitals and nursing homes remains unclear. Three research domains identified below (Supplementary Table 2 online) represent priority areas with unanswered questions concerning environmental disinfection in healthcare settings:

*Priority area 1:* What are the risks associated with environmental contamination with SARS-CoV-2 for HCP and patients?
*Priority area 2:* What are optimized methods for identifying environmental contamination with SARS-CoV-2?
*Priority area 3:* Determine the optimal methods for disinfection of healthcare environments.

Defining the risk associated with surface transmission is essential in assessing the potential benefit of decontamination and disinfection strategies. Although previously published studies demonstrate potential for fomite transmission, additional studies are needed to assess risk factors for both contamination and infection associated with contact with contaminated surfaces. In addition, these surface contamination studies were primarily cross-sectional studies and case reports using PCR detection of viral RNA on surfaces in COVID-19 units. Thus, while standardized methods have been proposed for evaluating surface contamination, evidence-based determination of optimal sampling strategies will enhance future work.^75^ Subsequent studies should include assessment of detection and correlation of findings with infectivity. Viral culture may be more useful in determining risk of infectivity, but there is inadequate infrastructure to broadly expand study of environmental contamination using this method.^68^ Enveloped viral surrogates including mammalian viruses and bacteriophage should be integrated into disinfection assessments. Establishing the infrastructure to define risk of surface contamination for other high-consequence pathogens is needed for future pandemic preparedness.

If surface transmission of COVID-19 is described, then reducing this risk within healthcare settings is necessary to provide care for vulnerable populations and to protect HCP. Implementation challenges can be substantial, but leadership support appears helpful. In a national study conducted in Thailand in 2014, Apisarnthanarak et al^76^ found that good-to-excellent hospital administration support for the infection control program was significantly associated with greater adherence to implemented environmental control and disinfection protocols. Thus, identifying the incremental benefit of enhanced disinfection strategies, such as ultraviolet germicidal irradiation, vaporized hydrogen peroxide, and others compared to commonly available disinfectants, will inform routine disinfection practices.^72,77^ The methods for evaluating the impact of such disinfection strategies should focus on clinical outcomes and laboratory methods that predict infectivity.

## 7. Drug and medical supply-chain shortages

The supply chain for drugs and medical supplies is global; most drugs and medical supplies are made outside the United States.^78^ Early in the pandemic, there were significant shortages of PPE, ventilators, and materials needed for laboratory detection of COVID-19.^79^ Drug shortages are situations in which patients are unable to access clinically interchangeable versions of regulated prescriptions due to supply limitations.^78^ Over the last decade, the number of drug shortages has increased dramatically.^80,81^ COVID-19 revealed consequences: manufacturers closed, governments prohibited drug and supply export, and patients and organizations stockpiled drugs.^29,82–84^ Of drugs consumed in the United States, 90% of raw active ingredients (active pharmaceutical ingredients [API]) are made in foreign facilities, 80% in China and India).^78,85^ Thus, supply-chain shortages are a complex global issue and can be influenced by geopolitical issues, trade, civil unrest, weather, and pandemics.^82,86^ The full extent of the impact of the COVID-19 pandemic, however, on the drug and medical supply chain is unknown. It is important to recognize and understand disruption in these supply chains to prepare for future pandemics and other global emergencies. Three research domains identified below (Supplementary Table 3 online) represent priority areas with unanswered questions concerning drug and medical supply shortages:

*Priority area 1:* Define the extent of drug and medical supply chain shortages caused, directly or indirectly, by the COVID-19 pandemic.
*Priority area 2:* Identify methods to disseminate best practices in order to optimize patient care in the face of drug and medical supply chain shortages, from local protocols to international policies to help mitigate future shortages.
*Priority area 3:* Characterize clinical consequences of drug and medical supply-chain shortages.

Recognizing disruption that can occur as a result of drug and medical supply-chain disruption, legislation included in the March 2020 “Coronavirus Aid, Relief, and Economic Security (CARES) Act” requires drug manufacturers to report the anticipated duration and the problem leading to a shortage.^80^ However, studies on the extent of these disruptions and effectiveness of such policies during COVID-19 and after the pandemic is unknown.

Also unknown are the serious outcomes related to these shortages, including worsening illness and premature death.^87–89^ In addition, little is known about how drug shortages impact clinicians who prescribe medications to treat critically ill patients. HCP reported anxiety about their inability to provide competent and evidence-based care during COVID-19.^60^ Minority populations and vulnerable populations may be disproportionately affected by these shortages. Drugs and other supply shortages may pose ethical dilemmas when decisions must be made to treat one patient over another.^90^ The impact of shared decision making and triage committees, which remove the responsibility from the individual provider, on HCP mental health and resiliency should be systematically evaluated.^29,60,90^

Addressing these research questions related to supply-chain disruption and the impact of drug and medical supply shortages will help preparations for future global emergencies and will inform national and international policies aimed toward decreasing the impact of drug shortages on patient outcomes.

## 8. Antimicrobial stewardship

COVID-19 caused a rapid shift in the delivery of health care, including the suspension of elective procedures and transition of in-person visits to virtual encounters.^91,92^ Changing health care delivery may lead to an increase or decrease in antibiotic prescribing, depending on the setting and patient population. Three research domains identified below (Supplementary Table 3 online) represent priority areas with unanswered questions concerning antimicrobial stewardship in healthcare settings:

*Priority area 1:* Identify the impact of changes in healthcare utilization and delivery on antibiotic prescribing.
*Priority area 2:* Define epidemiology and risk factors related to bacterial and fungal coinfections in patients with COVID-19.
*Priority area 3:* Develop and implement optimal antimicrobial stewardship program (ASP) strategies to improve antimicrobial use and patient outcomes while adapting to changing healthcare delivery during COVID-19.

Although a decrease in the number of admitted patients may lead to a reduction in overall antimicrobial use, several studies have suggested a large percentage of COVID-19 patients presumptively receive antibiotics to treat the potential that the infection is bacterial or that a superimposed bacterial infection is leading to a greater severity of COVID-19.^93–95^ Similarly, changes in the volume of healthcare access (decreased hospitalizations and outpatient visits) during the pandemic will limit longitudinal comparisons due to altered denominators for typical use metrics, patient bed days of care (for acute care), and in-person visits (for outpatient care). Additionally, with routine pediatric care transitioning to predominantly telehealth visits, the pandemic’s impact on outpatient antibiotic prescribing practices for children remain unexamined (Supplementary Table 5 online). Finally, the downstream impact of changes in antimicrobial prescribing on antimicrobial resistance and *Clostridium difficile* are unknown.

Typically viral respiratory infections have been associated with an increased risk of bacterial or fungal coinfections, which substantially increases risks of morbidity and mortality.^96–98^ However, data on coinfections in COVID-19 have been sparse and heterogeneous, with estimates ranging from 3% to 30%.^99–108^ To inform antimicrobial treatment, we must understand risk factors and timing for the development of coinfection. For example, the risk of coinfection related to comorbidity (eg, immunosuppression) or exposure (eg, hospitalization, ventilation, device-placement) is unknown.^97^ Furthermore, quantification of coinfection is limited by diagnostic difficulties including (1) distinguishing colonization from infection; (2) improving diagnosis of coinfection versus alternative causes of decompensation (eg, acute respiratory distress syndrome from COVID-19); and (3) limited respiratory culture data due to SARS-CoV-2 transmission concerns.^109^

The ongoing COVID-19 pandemic has drastically changed the way that ASP teams interact with patients and other healthcare providers.^110,111^ Although the focus of ASPs may have shifted during the pandemic, improving antimicrobial use and stemming the tide of the development of antibiotic resistance remain at the core.^110,111^ ASP teams are now ubiquitous in US hospitals, but ensuring that all hospitals have adequate infectious disease expertise on their teams may require novel approaches.^112^ Simultaneously, the responsibilities of ASPs have increased—with many playing an active role in COVID-19 management, such as guideline development or remdesivir allocation.^113,114^ Virtual strategies, such as ‘tele-stewardship’ and nimble regional or national ‘hotlines,’ particularly for small and rural hospitals, need to be evaluated and implemented. Likewise, although some nursing homes have established comprehensive nursing home ASPs, a required condition of participation by the US Centers for Medicare and Medicaid Services (CMS), many would benefit from additional support.^95^

## 9. Healthcare personnel safety and occupational safety

The COVID-19 pandemic has raised concerns about HCP safety. Research is needed to identify strategies to protect HCP from acquiring SARS-CoV-2 at work and to support HCP from physical, psychological, social, and organizational challenges related to the pandemic.^58,59,76^ Three research domains identified below (Supplementary Table 4 online) represent priority areas with unanswered questions concerning HCP and occupational safety:

*Priority area 1:* Define risks that increase HCP exposure to and acquisition of SARS-CoV-2 and interventions that can mitigate these risks.
*Priority area 2:* Determine optimized strategies to protect HCP emotional and psychological health.
*Priority area 3:* Determine impact of social and organizational strategies to maintain the health and wellness of HCP.

Understanding the factors that increase HCP risk of acquiring COVID-19 is essential to develop an evidence-based infection prevention program. These factors may include attributes of the patients under the HCP’s care (eg, clinical symptoms, comorbid conditions),aspects of the care delivered (eg, procedures performed, duration of contact, number of patients under their care during a shift, preoperative screening), HCP practices (eg, PPE utilized, years of experience), and work site (eg, leadership support, control over practice). In addition, understanding individual HCP factors that increase the likelihood of an infected HCP developing more severe disease and adverse outcomes will help determine which HCP may need additional protections in place, such as furlough, reassignment from the care of COVID-19 patients, or ongoing testing at a set interval during hospitalization. Research on costs and effectiveness of policies, such as preoperative and on-admission screening, can inform practice, improve HCP safety, enhance HCP confidence in safety processes, and reduce staffing challenges.^29^

Controversy persists about determining what medical procedures may allow opportunistic airborne transmission of pathogens traditionally considered to follow droplet transmission. Key considerations include the size of particles generated during specific procedures, and the ability of the pathogen to survive in small particles, and the infectious dose for the pathogen (amount of virus carried in small airborne particles sufficient to cause infection if inhaled). These questions have important implications for air handling and PPE selection recommendations to minimize transmission risk in healthcare settings.

Identifying policies that support the social, emotional, and economic needs of HCP is critical to maintaining workforce resilience and decreasing presenteeism, burnout, and turnover during the protracted COVID-19 pandemic.^60^ Developing an evidence base to inform these policies requires understanding how the pandemic has affected HCP social, emotional, and physical health, finances, ability to care for families, and decisions to come to work. A mixed-methods implementation science approach can inform local, institutional, and national policies and practices to support HCP resiliency, job security, ability to isolate or quarantine effectively, and social support (eg, hazard and sick pay policies, housing options, and consistent childcare).^106^

## 10. Return to work

Recommendations for return to work following COVID-19 infection are largely derived from experience with other communicable diseases such as influenza and norovirus. COVID-19 has posed specific challenges in timely return to work strategies due to minimal data on the true transmission dynamics and nature of exposure risks during HCP–patient or HCP–coworker interactions. Little is known about which HCP roles or activities confer the highest risks of transmission and how this risk is modified by severity of prior illness or presence of lingering symptoms upon return. Three research domains identified below (Supplementary Table 4 online) address the urgent priorities for research to facilitate timely worker return while tempering the risks of premature return to work during active illness:^115^

*Priority area 1:* Determine risk of SARS-CoV-2 transmission by returning HCPs to coworkers and patients, by HCP type and setting.

*Priority area 2:* Determine the optimal criteria and modifications necessary for earliest safe return to work.

*Priority area 3:* Determine the sociocultural impact of and strategies for successful return-to-work for HCP.

As of November 2020, the US CDC recommends that HCP who have had COVID-19 return at 10 days from initial symptom onset (20 days if immunocompromised) if improved and fever-free for 24 hours without fever-reducing medications.^116^ During a pandemic, sick leave of this duration, even when asymptomatic, could significantly impact healthcare system staffing. Many employers are considering the use of test-based strategies to shorten the window for HCP to return to work. Shared anecdotal experiences suggest a broad range of approaches for return to work across institutions and locations (eg, length of furloughs, strategies to address persistently PCR-positive recovered workers, coworker education, and acculturation to mitigate social stigma).

The true risk of virus transmission to coworkers or others from asymptomatic or minimally symptomatic HCP to patients or other coworkers remains largely unknown. Research is needed to assess the relative importance of worker-related factors, such as viral viability in minimally symptomatic or immunocompromised HCP, effectiveness of PPE or physical barriers to mitigate transmission, and risk of acquisition among immunocompromised coworkers and patients. HCP workflow, social culture, and nature of coworker interactions may also affect the likelihood of transmission within the workplace. Finally, research addressing worker reintegration and actions to assuage social stigmas (eg, educational needs of workers, childcare) are vital to retaining a talented and prepared workforce.^90^

## Conclusion

The SHEA COVID-19 research agenda is critical and ambitious. COVID-19 has exposed dangerous gaps in our understanding of the epidemiology, transmission, and individual as well as public health consequences of viral diseases. Global impacts on health, the economy, and progress have been felt in every population and country. The disease has disproportionately affected older adults, especially those living in nursing homes or long-term care facilities, racial minorities, and those with multiple comorbidities. Supply shortages have affected the health and well-being of HCP and have negatively affected care of those not infected with COVID-19. A well-planned, collaborative, comprehensive research agenda with careful, dedicated, and timely execution is a critical element to address the most important questions to more effectively limit outbreaks and pandemics. With the recognition that pandemics do not respect boundaries or economies, close collaboration between various disciplines is crucial. Research initiatives and trust between industrialized and developing nations are needed to address these critical questions in ways generalizable around the globe, including attention to capacity building, technology transfer, training resources, and aligning surveillance and prevention activities. This research agenda is a snapshot in time during the worst days of the COVID-19 pandemic and will certainly shift as the pandemic evolves and as vaccines and other therapeutics become available. Nonetheless, several priorities outlined have relevance to future infectious disease outbreaks and epidemics.

This research agenda calls for translational studies from laboratory-based basic science research to well-designed, large-scale studies and health outcomes research. To undertake this work, funding organizations must make COVID-19 research their highest priority. We anticipate that the next decade will be crucial in developing the next generation of epidemiologists, IPs, researchers, and leaders.

## Supplementary material

For supplementary material accompanying this paper visit http://dx.doi.org/10.1017/ice.2021.25. click here to view supplementary material
