# Supplementary material for: Coronavirus disease 2019 (COVID-19) research agenda for healthcare epidemiology
Source: Infect Control Hosp Epidemiol. 2021 Jan 25:1–11. doi: 10.1017/ice.2021.25 (PMC8160487; doi:10.1017/ice.2021.25)
Supplement: Supplementary file 1 [file S0899823X21000258sup001.docx]

**Supplemental Table 1: Epidemiology of the Disease, Outbreak Investigation, Surveillance**

| **PRIORITY AREA** | **RESEARCH QUESTION** | **CLINICAL RATIONALE** | **SUGGESTED METHODOLOGY/**  **POPULATION** | **INTENDED OUTCOMES** |
| --- | --- | --- | --- | --- |
| **EPIDEMIOLOGY** | | | | |
| **1. Heterogeneity in Transmission Dynamics** | 1a. How common are “superspreaders” and how do they impact the dynamics of the epidemic? | Transmission within the community/hospital/NH drives rates of infection. Better understanding the implications of “superspreaders” on transmission and basic epidemiological concepts such as R0/R(t), can better inform prevention practices as well as hospital utilization and clinical and HCP practices within the hospital | Observational studies of transmission by tracing outbreaks and using genetic tracing of infection to better understand the potential for nosocomial spread. | Characterize and prevent ‘superspreader’ events |
|  | 1b. What is the relative role of droplet vs. aerosol transmission, and how does this impact both models and observational studies of COVID-19? | This information has tremendous importance for public health initiatives (e.g., what types of businesses should be closed, when should people wear masks).  In the hospitals and NHs, this information affects the type of PPE needed, ventilation decisions, as well as management of patients (e.g., negative pressure rooms, isolation) | Prospective air sampling for viral particles, regular swabbing of surfaces for viral particles, and investigation of nosocomial transmissions in the hospital to identify potential relative risk (could be combined with daily swabbing of individuals in non-COVID-19 units to identify potential transmission events). Studies should assess the infectivity of recovered viral particles. | Evidence that can inform policy guidance at levels ranging from a single hospital or NH to the population at large. |
|  | 1c. Are infections within the hospital and NHs and among HCP primarily driven by transmission within the community or is there ongoing transmission in the hospital? Does this knowledge gap negatively impact HCP perception of risk and resiliency? | Understanding the source of HCP infections is critical to informing efforts to protect them and patients. Spending time and resources on interventions in settings that are not the primary source of transmission are unlikely to be impactful.  Awareness of when transmission risk is greatest (face-to-face contact, aerosolizing procedures, community interaction) can return control of practice to individual providers, which is a major driver of engagement and important for avoiding burnout and enhancing resiliency.^1,2^ | Contact tracing and other “shoe-leather epidemiology” conducted in conjunction with hospital epidemiology to fully capture the potential transmission chains that lead to a HCP infection.  Qualitative methods to understand relationship between transmission knowledge, personal risk perception, and use of protective measures (at work and at home) to capture knowledge in action and the impact on resiliency. | Estimates of how commonly HCP are infected during the course of patient care, and interventions that can appropriately address risk perception and incidence. |
| **2. Role of Asymptomatic / Presymptomatic Transmission** | 2a. How important are asymptomatic individuals to the epidemiology of SARS-CoV-2? | Following the transmission of disease and controlling spread in the community and the hospital requires identification of all infected and infectious individuals. However, while there appears to be a fraction of the population that is asymptomatic (as opposed to pre-symptomatic), uncertainty remains regarding the relative importance of these individuals to disease spread, their clinical course and immunity from future infection. | Cohort studies to show effectiveness of testing through PCR (possibly through a pooled approach), antigen detection, and serology to identify potential carriers and assess their contribution to transmission as well as to identify individuals already infected that are potentially immune and to follow them over time to identify their relative risk of infection. | Enhance understanding of the potential transmissibility, optimal identification methods, and resistance to further infection from asymptomatic individuals. |
|  | 2c. What interventions are most effective at controlling spread when individuals are presymptomatic/asymptomatic? | While increased testing may be useful for greater identification of asymptomatic individuals, uncertainty remains as to whether it is most cost-effective. Triggers to initiate public health initiatives and test their effectiveness. Do certain settings (e.g., hospitals, NHs) require different strategies? | Mathematical models of interventions that take account of heterogeneous transmission.  Observational analysis of intervention efficacy across counties/States in the U.S.  Assessment of point-of-care rapid tests to identify asymptomatic cases. | Policy recommendations and resource allocation for different jurisdictions and institutions, depending upon prevalence and function |
| **3. Risk Factors for Severe Disease Outcomes** | 3a. What demographic factors and clinical comorbidities are associated with severe COVID-19 disease outcomes? | Disparities in COVID-19 disease severity have been identified by age, race/ethnicity, gender, socioeconomic factors, and underlying comorbidities.^3^ Other disparities, such as disability status, may exist, but these have not been fully explored. HCP have become severely ill and thousands have died in the first months of the pandemic. Studies that assess multiple risk factors in combination are needed to enhance healthcare capacity and to characterize occupational risk of HCP. | Large-scale observational studies, including cross-sectional studies as well as retrospective case-control studies among those identified as infected with SARS-CoV-2.  Severity of disease can be modeled by individual and combined risk factors to understand the relative contribution of each that lead to worse outcomes. | More complete understanding of these demographic and clinical factors can inform public health interventions to reduce disparities, guide clinical case management and follow up, and help in assessment of occupational risk for HCP. |
|  | 3b. How does infectious dose at exposure affect severity of disease? | Specific settings (e.g., crowded, indoor spaces with poor ventilation) and healthcare procedures (e.g., aerosol-generating procedures) have been determined to be high risk because of the potential for higher-dose exposures. Cases of otherwise healthy HCPs acquiring severe disease suggest a possible effect of infectious dose. | Retrospective studies characterizing exposure or prospective cohorts of individuals with high occupational exposure risk to examine the relationship between types of exposure and severity of disease with dose-response analyses. HCP cohorts could be particularly informative. Animal studies of dose response. | A more complete understanding of the impact of infectious dose could inform recommendations to reduce exposure risk, such as avoidance of specific activities or settings and use of appropriate PPE in community and healthcare settings. |
|  | 3c. What are the long-term clinical sequelae of acute infection? | Evidence to date suggests the potential for protracted limitations in lung function as well as other neurologic, cardiometabolic problems, and inflammatory disease. Symptoms and functional limitations may manifest and endure after the initial acute phase of the disease. Their prevalence is not fully understood because of the relatively early timeline of the pandemic. In children there is particular concern due to Multisystem Inflammatory Syndrome in Children (MIS-C) associated with prior COVID-19 infection. | Longitudinal cohort studies that recruit and retain individuals with SARS-CoV-2 infection. Baseline and follow-up surveys at frequent intervals should be conducted to document clinical, psychological, and functional burden of down-stream impact of infection both for those with severe disease and those with mild/asymptomatic infections. | Understanding of full impact of the disease, including risk for chronic disability related to sequelae of disease and clinical practice guidance for long-term management, including implications for HCP who are infected in the course of providing care. |
|  | 3d. What is the role of immune response in protecting against severe disease and reinfection? | Immunocompromised individuals may shed infectious virus for longer duration, as reflected in updated guidance. The duration of immune response is not known, nor is there a robust evidence base about factors that predict strong immune response | Prospective cohort studies involving active surveillance for symptoms, PCR testing for viral load, and serologic testing | Could build evidence-base to inform updated isolation guidance and elucidate risk factors for re-infection and vaccine effectiveness, including the role of initial immune response in protecting against, or worsening the impact of reinfection (e.g., via antibody dependent enhancement) |
| **OUTBREAK INVESTIGATION** | | | | |
| **1. Outbreak Cessation** | 1a. What are the critical interventions required to stop an outbreak in acute care hospitals, NHs, and rehabilitation facilities?  Subtopics:  -education  -early identification  -public health involvement  -disease transmission  -social network analysis  -molecular sequencing  -environmental sampling  -geospatial mapping  -PPE – (respirators?)  -environmental management  -staff testing & management  -patient testing & management  -leadership | We need more targeted outbreak response to COVID-19, which involves many aspects of understanding local resources, local context, disease transmission, and utility of various response tools in various healthcare settings. | Meta-analysis of reported outbreaks and interventions.  Modified Delphi expert review of outbreak scenarios. | Improve COVID-19 outbreak response (investigation and mitigation); develop new and update existing Toolkits for outbreak response for use by healthcare facilities and local health departments. |
| **2. Personnel and Skillset** | 2a. What is the educational need, particularly in smaller hospitals and NHs without an infectious disease physician or healthcare epidemiologist, to optimally conduct an outbreak investigation?  2b. What are the potential roles of physicians and other staff not previously trained in infection prevention and control during an outbreak in small facilities with limited resources? | The capacity of facilities to rapidly identify, evaluate, and conduct an outbreak investigation is uncertain across health care. | Survey | Inform public health about the needs of diverse health care facilities in this regard. |
|  | 2c. What is the impact of COVID-19-related crisis management and outbreak investigations, in addition to routine infection prevention activities, on IP mental health, resiliency, and intent to leave the profession? | Gain insight into the effects of prolonged crises on infection prevention departments | Survey | Develop mitigation strategies to decrease loss of infection prevention department professionals |
| **3. Resources and Tools** | 3a. What resources are available and what is needed for hospitals and NHs to support outbreak investigation, including local public health capacity? | The resources available to facilities before and during an outbreak (education, personnel, leadership, lab capacity, software/ hardware) are uncertain. | Survey | Inform public health about the needs of diverse health care facilities in this regard. |
|  | 3b. Should real-time molecular sequencing be used to support an outbreak investigation? | If this technology were available and useful to support outbreak investigation, it would be important to include it in the standard approach. | Expert review of outbreak scenarios with and without sequencing. | Help inform public health and outbreak response teams to best determine if and when to apply this emerging technology |
|  | 3c. What type of social network analysis best supports an outbreak response? | Understanding the social interactions between individuals in hospitals and NHs and their care staff and therapists. The best way to gather and apply that information is uncertain. | Systematic review, modelling studies.  Comparatively apply various social network programs to outbreak situations. | Help inform public health and outbreak response teams to best determine if and how to incorporate social network analysis tools or software packages to outbreak scenarios. |
|  | 3d. What is the potential role of environmental sampling during an outbreak and the best type of lab (clinical vs. environmental) to obtain and analyze samples? | Understanding if and how environmental sampling could improve outbreak support. | Systematic review, modelling studies.  Expert review of outbreak scenarios with and without environmental sampling data | Help inform public health and outbreak response teams to best determine if, how, and when to apply this emerging technology. |
|  | 3e. Are existing Toolkits, including the SHEA/CDC ORTP Toolkit, effectively guiding outbreak response? | Are existing Toolkits useful and in what settings? | Survey; evaluation of the implementation of Toolkits during outbreaks in diverse settings (academic system, small hospital, LTCF) | Evaluate utility of existing Toolkits for all levels of practitioners likely to need this guidance |
| **SURVEILLANCE** | | | | |
| **1. Detection Strategies** | 1. Optimizing detection strategies of true COVID-19 burden | COVID-19 PCR can be positive for prolonged periods of time, up to 3 months. Need to differentiate between active and recovering cases. | Large-scale longitudinal surveillance studies with subgroup evaluation by symptoms (asymptomatic, mild-moderate symptoms, hospitalized patients). | Evaluate duration of PCR-positivity, antigen and seropositivity, risk for COVID-19 reinfection, and transmissibility |
| **2. Population-level transmission risks** | 2. Evaluation of high-yield test-based population surveillance strategies | Test sensitivity and specificity against assay controls may be high but clinical sensitivity and specificity for COVID-19 infection is relatively unknown and reported to be as low as 70%. | Clinical validity studies in  high vs low population prevalence states.  Compare PCR/serology against symptom based surveillance for measuring incidence and prevalence  Validity studies to evaluate sentinel surveillance strategies for large populations through en-masse testing – e.g., pooled saliva samples testing or sewer lines sampling. | Understand clinical performance characteristics of COVID-19-testing tools |
| **3. Population surveillance** | 3. Identification of high risk populations for targeted interventions | Defining exposure risks for individuals and groups through novel surveillance tools (e.g., personal exposure monitors, tracking apps) will guide the development of effective interventions. | Large-scale surveillance studies on a wide variety of community and work settings and activities associated with high risk for transmission  Specific attention needed in acute and LTCFs, factory/food processing plants, and places of education such as schools, colleges and universities. | Optimize prevention strategies and PPE use in a variety of healthcare and community settings. |

Abbreviations: CDC, Centers for Disease Control and Prevention; COVID-19, coronavirus disease 2019; HCP, healthcare personnel; LTCF, long-term care facility; MIS-C, Multisystem Inflammatory Syndrome in Children; NH, nursing home; ORTP, Outbreak Response Training Program; PCR, polymerase chain reaction; PPE, personal protective equipment; SARS-CoV-2, severe acute respiratory virus 2; SHEA, Society for Healthcare Epidemiology of America.

**Supplemental Table 2: Isolation Precautions, PPE, Environmental Disinfection**

| **PRIORITY AREA** | **RESEARCH QUESTION** | **CLINICAL RATIONALE** | **SUGGESTED METHODOLOGY/**  **POPULATION** | **INTENDED OUTCOMES** |
| --- | --- | --- | --- | --- |
| **ISOLATION PRACTICES** | | | | |
| **1. Initiation** | 1a. When should we initiate isolation precautions for:  a-acute care patients?  b-NH residents?  1b. How effective are current symptom-based screening measures in predicting COVID-19 for:  a-inpatients?  b-outpatients?  c-pre-procedural?  1c. Is symptom-based screening predictive of positive COVID-19 testing? | COVID-19 has a wide variety of clinical presentations ranging from asymptomatic to severely ill.  Further assessment of various clinical parameters could help optimize isolation initiation. | -Prospective observational studies in acute care hospitals and NHs, use of symptom diaries in outpatient settings  -Case Control studies to record symptoms in patients who tested positive vs. tested negative | Sensitivity and specificity of individual signs and symptoms to initiate testing for COVID-19 and to place patients/residents under isolation precautions while awaiting results.  Development of a validated integrated metric/scale that includes key signs/symptoms and has high sensitivity and specificity for predicting COVID-19 infection |
|  | 1d. Can testing be a useful adjunct to identify potentially infectious persons and isolate them before they are symptomatic?  For example, 1) Is it a useful strategy to test all new health care inpatients for COVID-19 regardless of their symptoms? 2) How useful is testing all NH residents regardless of symptoms and at what frequency? 3) What are the potential side effects of testing all new patients or NH care residents? | Detection of asymptomatic and pre-symptomatic patients is key for stopping transmission of COVID-19 given the increased infectiousness during the asymptomatic or pre-symptomatic incubation period.  Evaluating testing parameters will inform strategies to prompt timely isolation to stop COVID-19 spread. | -Quasi experimental studies comparing interval test-based screening vs. symptom-based or exposure-based testing  -Economic/cost-effectiveness analyses | To prevent secondary transmission through earlier isolation via a cost-effective and safe screening strategy. |
|  | 1e. At what distance are infectious aerosols transmitted for patients with COVID-19 during aerosol and non-aerosol generating procedure?  1f. How infectious are asymptomatic, pre-symptomatic and symptomatic patients?  1g. And for how long? How long after patient leaves room are there infectious aerosols in room?  1h. What is the added benefit of negative pressure vs. private standard rooms with the door closed?  1i. How much additional protection is achieved with use of PAPR or N95 vs. surgical mask during care delivery in the clinical setting?  1j. What practices are needed to manage patients with aerosolizing procedures? Is it safe to cohort COVID-19 positive patients during surge capacity conditions? | Understanding transmission characteristics is critical for determining what type of PPE to use for routine clinical practice and aerosol generating procedures; the need for airborne precautions; and, the cleaning/disinfection process for room turnover. | -Basic science research  -Retrospective analysis correlating symptoms with diagnosis of secondary cases. This would include long term care and during outbreak /exposure investigations | To improve patient and healthcare personnel safety and determine effective high impact vs. low impact interventions. |
|  | 1k. How do PPE shortages and insufficient or unclear guidance affect clinical care and healthcare personnel well-being? Are there specific populations that are impacted more than others? How do the internet and social media exposure and the phenomenon of infodemia (enormous unfiltered information) impact feelings of insecurity and anxiety by HCP?^4^  1l. Do HCP working on the frontlines (e.g., with COVID-19 patients) have different stress and anxiety than HCP providing supportive roles (e.g., non-COVID-19 units, infection preventionists, emergency departments)^2^ | Rapidly evolving guidance and PPE shortages can impact healthcare personnel well-being, turnover, patient care and health disparities.  Early evidence suggests those on the frontlines may feel “closer to key decision makers and have had access to more timely and accurate information”, thereby lessening stress and anxiety.^2^ Targeting those at greatest risk for misinformation and resultant stress and anxiety can streamline intervention efforts. | Mixed methods studies of patients, healthcare providers including minority populations and public health officials | Characterize unintended consequences of rapidly changing guidance on patient outcomes and healthcare provider well-being.^2,4^ |
| **2. Management** | 2a. How do COVID-19 isolation precautions affect risk of developing healthcare-acquired conditions (e.g., pressure ulcers, delirium, functional decline, and catheter-associated infections) or other adverse outcomes (e.g., anxiety, depression) among:  1-acute care patients?  2-NH residents? | Defining risk and unintended consequences of isolation precautions in hospitalized patients and NH residents with COVID-19 is essential for ensuring safe care. | -Prospective cohort studies of COVID-19 cases in acute care and NH populations. | Timely identification of unintended harmful consequences of isolation precautions in populations with COVID-19 with the intent of developing strategies to reduce harm. |
|  | 2b. What are effective strategies, ideal work environments and cultural norms for optimizing how healthcare personnel interact with patients in COVID-19 isolation precautions in:  1-acute care?  2-NHs? | Identifying ways to safely provide care to COVID-19 patients while minimizing risk of transmission includes understanding and addressing various aspects of the work environment. | -Qualitative methods, including direct observation and time motion studies to understand and assess preparation for and in-room care provided by healthcare personnel  -Development and testing of human factors-based redesign interventions  -Mixed-methods approach to understand ideal work environments and leadership culture that supports adherence to isolation precautions | Define high risk procedures and care processes that take the most time or might lead to potential safety related concerns. Develop practical strategies to provide safe and effective care to patients and NH residents^5^  Identify cultural norms/ideal work environment/leadership actions that support HCP adherence to isolation precautions. |
|  | 2c. How can family and informal caregivers be safely involved in the care of patients, adult and pediatric, in COVID-19 isolation precautions, including in acute care, NHs, and assisted living facilities? | Family and caregiver restrictions can have detrimental effects, prompting a need for in-person and virtual strategies to incorporate patient families and caregivers in care of their loved ones affected with COVID-19. | - Prospective surveillance studies to evaluate whether families and caregivers have immunity to COVID-19  -Implementation studies to test innovative technologies | Identify ways to safely enhance patient and caregiver satisfaction in caring for COVID-19 patients. |
|  | 2d. What are effective strategies to ensure care is aligned with patient care goals and healthcare personnel actions in the context of COVID-19 isolation precautions? | Determining patient care goals at a time of intense stress and uncertainly is challenging. Thus, ways to ensure care is aligned with patient goals in the event of COVID-19 and other outbreaks from COVID-19 and other respiratory illnesses are needed. | -Patient-oriented and/or pragmatic trials of high-risk populations and their families | Provide goal-directed care during COVID-19 and other pandemics. |
| **3. Discontinuation** | 3. What are the most effective criteria for discontinuing COVID-19 isolation precautions in:  a-acute care?  b-NHs?  For example, 1) What is the risk of secondary cases with a testing vs. symptom/time-based approach? 2) What symptoms are associated with no transmission risk and could be used to discontinue COVID-19 isolation precautions? | Established criteria for isolation discontinuation are important for determining when certain activities can be safely started or restarted e.g., rehabilitation focused activities, group activities, while also reducing the risk of secondary transmission. | - Prospective or retrospective epidemiologic studies to determine rate of secondary transmission following discontinuation of precautions at different intervals or symptom resolution | Provide evidence-based guidance regarding discontinuation of isolation and precautions that balance public health and patient priorities. |
| **PERSONAL PROTECTIVE EQUIPMENT (PPE)** | | | | |
| **1. Appropriate PPE** | 1a. What is the appropriate level of universal PPE? | Understanding the benefit, unintended consequences, and specific components of universal respiratory viral PPE that minimizes HCP acquisition of COVID-19 is important. | Prospective studies such as with a cluster randomized controlled trial may be difficult; simulation studies may be preferred.  Suggested outcome measures:  1) efficacy of mask and eye protection strategies in preventing droplet transmission, 2) occupationally-acquired COVID-19 among HCP (rates per population), and 3) attitudes of HCP re: use, tolerability, barriers to patient care delivery with universal PPE. | Evidence based PPE recommendations  Adjustments, if possible, for higher-risk exposures (e.g. care for known COVID-19 patients, community and household exposures) should be considered. |
|  | 1b. At what point do respirators lose their effectiveness? | In times of PPE shortage, extended use and reuse of PPE intended to be single use may become necessary. Data are scarce about safe strategies in these situations, including with duration of wear and the count of times donning or doffing. | Assess PPE durability and persistent efficacy ideally in laboratory settings that can assess material components filtering efficacy in usual use scenarios, durability of those components in experimental settings, and then lastly in clinical use by HCP. For example, new isolation masks or N95s could be tested for filtration effectiveness using standardized criteria before use, after short term/single use, after a several donning and doffing events, after a full day of use, after use and storage in different conditions, and after multiple days of use.  Suggested outcome measures: Material integrity and filtration efficacy in terms of particle capture, both inbound (to the wearer) and outbound (from the wearer), by particle size and by usage case tested (e.g. number of donning/doffings, hours continuous use, storage duration). | Evidence based recommendations for PPE conservation strategies that maximize HCP safety |
|  | 1c. Where do errors occur in donning and doffing of PPE? How can appropriate donning and doffing be optimized? Do errors in donning and doffing PPE play a major role in SARS-CoV-2 transmission? | There has been increased attention to PPE use practices since the Ebola outbreak in 2014. This has resulted in the further development of HFE work designed to identify key aspects of PPE donning and doffing protocols that may impact the likelihood of HCP self-contamination. The COVID-19 pandemic provides an opportunity for careful assessment of current PPE use practices to identify the most common errors in donning and doffing, and to identify the barriers and facilitators to effective PPE use. | HFE and mixed-methods approaches would be used. Structured observations of PPE use at several different centers on COVID-19 units could be conducted to identify hazards in HCP donning and doffing protocols. Investigators could collect and compare PPE use protocols across different institutions, comparing them with CDC and WHO guidelines. Suggested outcome measures: Rates of errors in donning and doffing of PPE, variation in error rate by different steps in PPE donning and doffing, deviations in local PPE guidance from CDC and WHO guidelines for PPE donning and doffing, barriers and facilitators to PPE use, and identification of concerns and issues with PPE donning and doffing using structured interviews with HCP. | Improved recommendations for safe PPE donning and doffing. |
| **2. Strategies to improve PPE** | 2a. Do face shields provide any incremental benefits to masks? | Face shields, used as a form of eye protection, are used as an added barrier over a face mask (both surgical masks and particulate respirators) with the rationale that this may prevent exterior contamination of the face mask. | Testing for reduction in external mask contamination could involve collection of face masks used in the clinical care of confirmed COVID-19 patients with and without face shields as the method of eye protection.  Considerations for adjustment by use during the performance of aerosol-generating procedures should occur as this would be expect to increase the risk of external contamination of the face mask.  Primary outcome would be the recovery of SARS-CoV-2 from the exterior of face masks. Unintended effects of wearing the face shield over other forms of eye protection (e.g. impairment of visual acuity on precision tasks) could be assessed by provider survey. | Evidence based recommendations on use of face shields for COVID-19 protection |
|  | 2b. Are there differences in protection provided by different types of respirators: (PAPR vs N95 vs KN95)? | Clinicians often state they think PAPRs, or elastomeric respirators provide enhanced protection against small particle aerosols compared to N95 or KN95 respirator masks although they have similar indications for use in healthcare settings. Data on particle filtration with each respirator during healthcare delivery would provide useful information for risk assessment. | Testing of filtration efficacy of various respirator types could involve use of biologically inert small particle size deployed in experimental settings where tracer can be measured before and after the respirator and in simulation settings while the respirator wearer simulates the movements of providing healthcare during potentially high risk procedures. Tracers should be measurable by deposition on the inside of the different respirator types. Ideally testing would be done with a range of respirator size and brands and a range of wearers of different age, weight, and gender.  Outcomes would include tracer detection inside/past the respirator by respirator type across wearer variables. | Evidence based recommendations around preferred respiratory protection for airborne isolation |
|  | 2c. What are the sociobehavioral, contextual, and adaptive factors required to improve appropriate PPE use? | Appropriate use of PPE in healthcare settings can substantially reduce transmission risk. However, PPE guidance may not be universally followed. Understanding how to improve appropriate PPE use, through understanding the sociobehavioral, cultural, and adaptive reasons for not following appropriate PPE guidance, could improve PPE adherence among healthcare workers. | Mixed methods studies would identify specific sociobehavioral, cultural, and adaptive barriers that contribute to compliance with PPE guidance in healthcare settings, including qualitative methods such as stakeholder interviews and observations. Implementation studies using a differences in differences approach could study the impact of interventions to reduce barriers to PPE compliance. | Outcomes of qualitative or observational work may produce an understanding of which sociobehavioral, contextual, or adaptive factors are required to improve appropriate PPE use. |
|  | 2d. What is the appropriate level of universal PPE (for pandemic and for future state)? What is the additional benefit that face shields may provide to the underlying face mask? | COVID-19 is thought to primarily spread through droplets, thus the current role of PPE is aimed at decreased droplet transmission. Masks are used as the cornerstone of source control but it is possible that COVID-19 transmission can occur through the eyes, either by direct droplet inoculation or via auto-inoculation. Data on the role of the appropriate level of PPE is needed. | Methods could include before/after studies regarding acquisition of COVID-19 with or without face shields, natural history studies of COVID-19 and flu rates before/after universal masking, and HCP surveys.  Primary outcome would be development of COVID-19 after exposure. Other outcomes could be more qualitative including comfort while wearing PPE, HCP perceptions of PPE, and ability to perform work function utilizing PPE. | Evidence based, user informed recommendations for universal PPE. |
|  | 2e. Will the overwhelming number of seriously ill patients that required isolation, the shortage of PPE, and the evolving information regarding disease transmission risks impact adherence to PPE practices in the short and long term? If so, how? | Factors impacting adherence to PPE use among HCP are unclear, but may be impacted by the rapidly changing nature of the pandemic. In particular, local factors such as surge in patients requiring isolation, ability to acquire PPE, changing guidance on PPE use, and evolving data on disease transmission risks may impact adherence to PPE practices over time. | Methods could include structured observations of PPE use at several different centers on COVID-19 and non-COVID-19 units over time and with different timing of patient surges or ability to acquire PPE, with a focus on what PPE is used. In addition, surveys could be done over time to focus on attitudes towards and practice of PPE use.  The primary outcome could be changes in PPE use over time. | Evidence-based recommendations for providing HCP with PPE guidance in the setting of rapid changes. |
| **3. Impact on other viruses** | 3. Are interventions made (specifically PPE) during the COVID-19 pandemic likely to be effective against other commonly-circulating respiratory viruses? | There is already anecdotal evidence that some areas in the Southern Hemisphere are seeing dramatic reductions in influenza during their annual seasonal flu time period. Widespread adoption of COVID-19 prevention strategies (physical distancing, face coverings, limitation on indoor gatherings, travel restrictions) would also prevent spread of other viral respiratory pathogens. Such a trend may also be seen within healthcare facilities as we move to a form of “universal droplet with eye protection” for almost all patient care activity, and universal source control for other hospital areas (primarily masking). | A large retrospective multicenter study to gather data from across multiple hospitals to assess rates of healthcare-associated respiratory viral illness before and after each hospital implemented universal source control strategies for COVID-19 as recommended by CDC.  Commercial respiratory viral panel data could allow both community and healthcare associated infections.  Suggested outcome measures: 1) rates of healthcare-associated influenza, RSV, or other infections (test performed >3 days into hospitalization); (2) rates of test positivity across respiratory viral panels regionally or nationally, possibly paired with data on date of test performance compared to date of admission (for those performed on inpatients); and (3) influenza and influenza-like illness data as currently gathered by public health authorities, as a window into community infection incidence. | Evidence-based improved recommendations for PPE for other respiratory viruses |
| **ENVIRONMENTAL DISINFECTION** | | | | |
| **1. Risks related to environmental contamination** | 1a. Does viral contamination of surfaces increase risk of patient or healthcare personnel infection with SARS-CoV-2? | Significance of indirect transmission via contaminated surfaces with SARS-CoV-2 is unknown | Animal studies to investigate risk for indirect infection via contaminated surfaces | Approximation of the risk that indirect transmission poses to healthcare workers and uninfected patients |
|  | 1b. Do certain patient characteristics, patient care procedures, or healthcare settings predict greater risk of environmental contamination of surfaces? | Degree of environmental contamination may change in disease course or related to procedures/support provided to patients | Longitudinal comparison of environmental contamination to identify risk factors | Determine risk factors for environmental contamination which may inform cleaning practices |
|  | 1c. Do specific surfaces such as shared medical equipment, or contaminated PPE, pose a greater risk for indirect transmission? | Shared equipment and fomites have been linked to transmission with other pathogens | Surveillance of contamination of shared equipment | Determine the risk of shared equipment for indirect transmission |
|  | 1d. Are long term care facilities associated with increased environmental contamination with SARS-CoV-2 compared with acute care facilities? | Long term care facilities typically have less robust air handling and air exchange than acute healthcare settings and may have less robust environmental cleaning/disinfection programs. | Repeated sampling of surfaces in long term care settings, including air vents and surfaces in communal areas. | Determine impact of the long-term care environment on persistence of SARS-CoV-2 on near patient surfaces. |
| **2. Strategies for evaluating environmental contamination** | 2a. Which laboratory methods for evaluating viral contamination of surfaces best predict both risk of infection and effectiveness of decontamination? | Significance of viral RNA on surfaces is unknown, both before and after cleaning | Investigation into the sensitivity of sampling techniques and the correlation of findings with potential infectivity | Evidence based methods for surveillance of environmental contamination and effectiveness of disinfection including comparison of PCR and viral culture |
|  | 2b. What is the optimal sampling method for recovery of SARS-CoV-2 from surfaces? | Optimal method (cotton vs swab vs sponge vs other) to recover viable SARS-CoV-2 from surfaces is not known. Each method likely has a different sensitivity for SARS-CoV-2 recovery. | Lab based study: spike surfaces with known quantities of SARS-CoV-2 or surrogate, sample using different methods to assess quantity of virus recovered by each method. | Determination of optimal sampling method for recovery of SARS-CoV-2 from surfaces. |
|  | 2c. Can viable SARS-CoV-2 be isolated from feces, and if so, at what frequency. If viable SARS-CoV-2 can be isolated from feces, can viable virus be aerosolized by incontinent patients and/or flushing a toilet? | The significance of fecal contamination is unknown | Study of fecal and urine shedding and contamination of surfaces near toileting areas | Determine the risk of environmental contamination associated with toileting |
|  | 2d. If viable SARS-CoV-2 can be isolated from feces, can viable SARS-CoV-2 be isolated from wastewater (sinks, drains, toilets)? | Presence of SARS-CoV-2 in wastewater or sewage may serve as a marker of local incidence, or a risk for acquisition | Study of wastewater reservoirs and adjacent surfaces to determine prevalence of contamination at these sites | Determine prevalence of wastewater contamination |
| **3. Disinfection strategies** | 3a. Do enhanced modalities for environmental decontamination such as UVGI or hydrogen peroxide vapor provide additional benefit compared to standard disinfection strategies? (#3) | These modalities have been associated with decreased transmission of other pathogens, but the incremental benefit to standard cleaning practices for SARS-CoV-2 is unknown | Surveillance of environmental contamination before and after cleaning stages using standardized laboratory methods | Determine the incremental benefit of such strategies to decrease risk of indirect transmission; secondarily determine cost-benefit ratio |
|  | 3b. Does increased frequency of cleaning of patient rooms and common areas significantly reduce surface contamination? | Increased frequency of cleaning of high touch surfaces is commonly performed, but the benefit has not been quantified. Similarly, few studies have focused on cleaning and disinfection strategies outside of patient rooms (e.g., break rooms) | Comparison of viral contamination of surfaces before and after cleaning events | Determine the benefit of increased frequency of cleaning for reducing environmental contamination within rooms and in common areas. Evaluate if practices designed to improve disinfection for SARS-CoV-2 secondarily improve disinfection for other MDROs. |
|  | 3c. Are long term care facilities associated with increased environmental contamination with SARS-CoV-2 compared with acute care facilities? | Long term care facilities typically have less robust air handling and air exchange than acute healthcare settings and may have less robust environmental cleaning/disinfection programs. | Repeated sampling of surfaces in long term care settings, including air vents and surfaces in communal areas. | Determine impact of the long-term care environment on persistence of SARS-CoV-2 on near patient surfaces. |

Abbreviations: CDC, Centers for Disease Control and Prevention; COVID-19, coronavirus disease 2019; HCP, healthcare personnel; HFE, Human Factors Engineering; NH, nursing home; PAPR, power air-purifying respirator; PPE, personal protective equipment; RNA, ribonucleic acid; RSV, respiratory syncytial virus; SARS-CoV-2, severe acute respiratory virus 2; UVGI, ultraviolet germicidal irradiation; WHO, World Health Organization.

**Supplemental Table 3: Drug and Medical Supply Shortages, Antimicrobial Stewardship**

| **PRIORITY AREAS** | **RESEARCH QUESTION** | **CLINICAL RATIONALE** | **SUGGESTED METHODOLOGY/**  **POPULATION** | **INTENDED OUTCOMES** |
| --- | --- | --- | --- | --- |
| **Drug and Medical Supply Shortages** | | | | |
| **1. Impact of shortages** | 1a. What is the extent drug and medical supply shortages during the COVID-19 pandemic? | Description of type, severity, and magnitude of shortages as well as the patient populations most highly impacted by these shortages is needed. | Cross-sectional and longitudinal studies of inpatient and outpatient populations | Through understanding the agents and populations most impacted, we can better target patients and/or health care settings at highest risk to prevent or mitigate future shortages. |
| **2. Dissemination of best practices** | 2a. What are the best local institutional, national, and international policies and strategies to optimize patient care during times of shortage and mitigate future drug and medical supply shortages? | Identifying effective policies to mitigate shortages during pandemics/states of emergency is essential to prevent future shortages. Individual patient-based and institutional strategies to conserve drugs and medical supplies on shortage have been implemented piecemeal during the pandemic. | Descriptive studies of policies and practices in response to drug and medical supply shortages and potential impact on supply chain | Identifying the most effective policies to decrease disruption in the drug and medical supply will assist in improving the supply chain and mitigate shortages. Sharing successful strategies and management practices can help improve patient outcomes and decrease the negative clinical consequences known to be associated with drug and medical supply shortages. |
| **3. Clinical consequences** | 3a. What are the downstream clinical outcomes of these drug and medical supply shortages? | Patient safety issues, adverse events, infection prevention and control, and increased morbidity and mortality are all potential negative consequences of drug and medical supply shortages. | Cohort studies of inpatient COVID-19 positive patients | Providing clinicians with insight and experience from colleagues also impacted by these shortages this will lead to improved understanding of potential negative clinical consequences and improved management strategies. |
|  | 3b. What is the impact of drugs shortages in management of COVID-19 and other associated chronic conditions? | Drug shortages of therapeutics with suspected effectiveness for COVID-19 have downstream impact such as the exhaustion of supply chain and medication hoarding, which has resulted in a shortage for patients chronically taking the medications and unavailability of drugs for hospitalized patients (e.g., hydroxychloroquine). | Ecological studies on groups of patients receiving these agents for chronic conditions and availability of these drugs at different times pre and during the pandemic | Assess shifting of drug supply and cost at the patient-level (e.g., shifting from chronic to acute use) and health care sector-level (e.g., shifting from outpatient to acute care use). Identify methods to maintain drug supply needed to maintain medication regimens for patients taking the medications for chronic use. |
|  | 3c. What are the economic consequences of the drug and medical supply shortages that have occurred during COVID-19? | Economic consequences of drug and medical supply shortages (drug costs, labor costs, medical supply costs, infection prevention costs, laboratory costs, overall institutional cost changes) | Longitudinal studies considering institutional cost impact of drug and medical supply shortages secondary to COVID-19 | The extent of economic impact of drug and medical supply shortages is poorly defined. The impact of drug shortages on patient out-of-pocket cost is unknown. Identify supply chain factors associated with rising costs (e.g., few manufacturers, generics). |
|  | 3d. What ethical dilemmas are associated with drug and medical supply shortages brought on by COVID-19? | Drug and medical supply allocation with limited supply and lack of alternative products raise issues of identifying patients who should receive a product with a limited supply (e.g., potentially lifesaving medication, laboratory test, ventilator). | Qualitative interviews and focus groups of health care professionals at health care facilities with patients with COVID-19. | Identification of processes to ethically allocate products with limited supply with health equity in mind. |
|  | 3e. What is the impact of changing infection control strategies on the development of drug and medical supply shortages for specific agents? | Changing infection prevention policies to decrease contact with COVID-19 patients have the potential to change prescribing patterns and PPE and lead to further shortages (e.g., changing from nebulized treatments to inhalers; extended infusions to decrease number of times nurses enter the room for drug administration). | Quasi-experimental studies of inpatient and long-term care populations to demonstrate change in drug and medical supply availability | Understanding the role of changing infection prevention strategies downstream on availability of specific drugs and medical supplies will assist in mitigating future supply issues and inform a national list of essential medicines and medical supplies. |
| **ANTIMICROBIAL STEWARDSHIP (ASP)** | | | | |
| **1. Healthcare utilization** | 1a. How have the changes in health care utilization and delivery affected antimicrobial prescribing? | As healthcare delivery changes, antimicrobial prescribing may change which could have long-term effects on antibiotic resistance. | Creation of novel metrics that account for changing patient census and prescriptions via non-traditional routes (e.g., tele-visits) to assess prescribing patterns, delays in diagnosis and/or delays in care. | Identify and quantify how antimicrobial prescribing has changed with the pandemic and inform future research into the effects of COVID-19 on antibiotic resistance and adverse events. |
| **2. Coinfection** | 2a. How common are bacterial and fungal co-infections in patients with COVID-19? | Patients with viral respiratory infections are often at increased risk of bacterial co-infection. Patients with COVID-19 also appear to be at increased risk for fungal co-infection. Data are limited on prevalence, risk factors, and outcomes for bacterial and fungal co-infection in COVID-19. | Cross-sectional and longitudinal studies evaluating prevalence and incidence of bacterial and fungal co-infection, and hospital acquired infections, in patients with COVID-19 a) in the community, b) on hospitalization, and c) in the intensive care unit/after exposure to additional risk factors. | Allow risk stratification to identify patients with COVID-19 who may benefit from empiric antimicrobial therapy. |
| **3. Effective Stewardship Strategies** | 3a. What are optimal ASP strategies to improve antimicrobial use and patient outcomes while adapting to changing healthcare delivery during COVID-19? | ASP is often resource intense and requires in-person presence. Increasing use of distanced or collaborative strategies may be able to more efficiently optimize antimicrobial use | Prospective, quasi-experimental, or cohort studies of stewardship interventions during COVID-19 that address the four moments of stewardship:  1) diagnosis, 2) cultures and empiric therapy, 3) stop/narrow/change to oral, 4) duration (and discharge).  Financial analyses and surveys on how ASPs have been affected by budget cuts. | Identify optimal treatment and stewardship strategies to improve antimicrobial use. Lessons learned about delivering stewardship remotely during the pandemic could inform efforts to expand access to underserved populations (e.g., rural, international, long-term care stewardship). |
|  | 3b. How have and should diagnostic tests be used to improve antimicrobial use and COVID-19 treatment? | Imperfect tests for COVID-19 and bacterial and fungal infection lead to diagnostic uncertainty which may delay treatment or trigger overtreatment (e.g., unnecessary antibiotic use). Improved diagnostic tests and data on strategies to optimize testing while minimizing cost are necessary to improve patient care. | Diagnostic test studies to improve our understanding of COVID-19 test performance, biomarkers, cultures, and non-culture tests.  Retrospective, quasi-experimental, and implementation studies of diagnostic stewardship strategies to improve test utilization, diagnosis, and treatment of COVID-19 and co-infections. | Improve diagnosis and management of COVID-19 while limiting unnecessary antimicrobial use and harm from over-testing (cost, blood draws, etc.). |
|  | 3c. How have and can ASPs help optimize the use of COVID-19 therapeutics (e.g. remdesivir), which may be in limited supply?” | ASPs often have diverse, multidisciplinary teams. The existing infrastructure (e.g., information technology) and collaboration could facilitate rapid, multi-faceted responses to the COVID-19 pandemic and other crises through resource stewardship of more than just antibiotics (e.g., non-antimicrobial drugs needed for treating COVID-19 patients, such as steroids, or other resources such as ventilators). | Descriptive case studies of hospital responses and frameworks for how to leverage existing infrastructure to battle urgent needs. | Create a model for future pandemic response and highlight the value of investment in stewardship to improve hospital preparedness. |

Abbreviations: ASP, antimicrobial stewardship program; COVID-19, coronavirus disease 2019.

**Supplemental Table 4: Healthcare Personnel Safety/Occupational Safety and Return to Work**

| **PRIORITY AREAS** | **RESEARCH QUESTION** | **CLINICAL RATIONALE** | **SUGGESTED METHODOLOGY/**  **POPULATION** | **INTENDED OUTCOMES** |
| --- | --- | --- | --- | --- |
| **HEALTHCARE PERSONNEL SAFETY/OCCUPATIONAL SAFETY** | | | | |
| **1. HCP exposure** | 1a. What is the relative risk of COVID-19 for HCP (considering both individual and occupational factors)? | Understanding incidence and risk-factors for HCP COVID-19 infection is essential for prevention of these events. | Epidemiologic study of HCP infection in comparison to a risk-matched community cohort. | Defined risk associated with providing healthcare services. Identification of risk-factors for HCP infection including occupation, exposures, and HCP health status. |
|  | 1b. What is the optimal approach to HCP exposure management (i.e. defining exposure, contact tracing, role of post-exposure testing, etc.) | Evidence-based classification of exposure events and the associated risk of COVID-19 infection is needed to prevent secondary infection while maintaining an effective workforce. | Epidemiologic study of exposure events and incidence of COVID-19 infection among HCP. Study of interventions to prevent transmission should adjust for community rates of infection. | Identified risk associated with common exposures in the hospital setting. Description of the effectiveness of exposure management practices with a cost-effectiveness analysis.  Consider qualitative approaches to investigate the impact of exposure management practices on mental health and moral injury of HCP.^6^ |
|  | 1c. What procedures generate infectious small particle aerosols that would require the use of a respirator, (and to what extent do those aerosols contain transmittable virus)? | Identifying clinical care procedures where respirator use is most needed may be helpful in mitigating risk to HCP in the setting of critical PPE shortages. | Experimental models to determine risk of aerosol generation associated with clinical care procedures. Observational studies of COVID-19 patients with air sampling to determine if replication-competent virus can be detected in aerosols after such procedures. | Determine risk associated with clinical care procedures that may generate small particle aerosols, to aid in the appropriate allocation of respirators. |
|  | 1d. What is the efficacy of periodic specific screening measures (temperature screening, symptom checks, testing of asymptomatic individuals, etc.) of visitors, patients, and HCP on the ability to protect the healthcare workforce? | Identifying those who may be in early stages of the disease who are contagious and may spread infection to others may limit exposure to all HCP and others in the hospital. | Retrospective case-control of HCP who participated in periodic screening and developed symptoms exposing others at work. Descriptive study of symptom onset and development of the presence of viral antigen in those who develop SARS-CoV-2.  Descriptive and retrospective case-control study of Veterans Affairs hospital policies and procedures, with focus on special/vulnerable population (spinal cord injuries and disorders) | Clear data that identifies prodromal symptoms that can reliably predict SARS-CoV-2 infection in HCP and visitors who enter the hospital.  Focus screening processes on those with significant predictive value. |
|  | 1e. What is the role of periodic testing of asymptomatic HCP for SARS-CoV-2 (viral antigen and antibody tests), without an identified exposure to a person with COVID-19, and how should this information inform staffing decisions? | Detecting those who are in the prodromal stage who are contagious is essential to limiting spread of the infection to HCP and others while in the hospital environment. | Prospective cohort study of asymptomatic HCP in hospitals and nursing homes for SARS-CoV-2 antigen and antibody.  Cohort study of staff SARS-CoV-2 infection within specialized units in VA such as spinal cord unit, community living centers, and palliative care units. | Prevalence of SARS-CoV-2 viral antigen and antibody in HCP who have not had a known exposure to a person with COVID-19. |
|  | 1f. What features of the physical work environment may reduce risk for HCP-to-HCP exposure (e.g. How do physical distancing measures and other physical barriers [e.g., Plexiglas] in the workplace affect HCP safety)? | Identifying the minimal physical intervention while maintaining safety in the workplace will decrease cost, increase staff and patient satisfaction and keep HCP and others safe. | Observational studies with air sampling to determine if replication-competent virus can be detected at several distances (e.g., 6, 9, & 12 feet) and the effect of various physical barriers on the ability to detect virus. | Reliable data on the detection of replication-competent virus at different distances with and without physical barriers. |
| **2. HCP Mental Health** | 2a. What has been the impact of the COVID-19 pandemic on HCP emotional and psychological wellness (both to the individual and in the delivery of patient care) and what interventions will help mitigate this impact? | HCPs have been noted to have high levels of stress, anxiety, and psychological distress. Multiple studies have demonstrated high levels of stress, anxiety and psychological distress in HCP during COVID-19.^2,7-11^ | Survey of HCPs centered around the impact of COVID-19 from a mental health standpoint, and dissemination and implementation studies focused on mental health resources, including mobile or virtual technologies.  Evidence synthesis is needed, with mapping to studies of military, disaster, and other traumatic exposure, to identify common themes and existing interventions to be implemented.{Rauch, 2020 #127}^12^ | Outcomes include changes in mental health over time, and before/after interventions.  Identification of an index to quickly detect the needs of a population and propose effective interventions in near-real time that are acceptable to the target group.^4,13^ |
| **3. Social/ Organizational Barriers and Facilitators** | 3a. What societal and organizational support/infrastructure/policies will facilitate maintaining an active, resilient healthcare workforce during the COVID-19 pandemic (e.g., childcare, homeschool for school-age children, family illness, hazard pay, living quarters, policies to maximize job security, etc.)? | The COVID-19 pandemic has caused multiple disruptions to the infrastructure surrounding a viable workforce. Without schools and daycares, it makes it difficult for HCPs to be able to work. HCPs are also putting themselves at risk for COVID-19, thus policies such as hazard pay, paid sick leave, and job security packages may help keep the workforce viable, and reduce presenteeism. | Surveys of HCPs to understand needs, and workplace interventions such as sick leave policies or hazard pay, before/after studies.^1^  Starting point for mixed-methods study development (survey-interviews) could be work by Shanafelt, et al, that outlines sources of anxieties among HCP during COVID-19.^1^  Suggested outcomes: Outcomes include ability for HCP to stay in the workforce, days of lost salary or days off work, burnout, turnover intentions, and general survey to understand needs of HCPs. | Evidence based strategies for organizations and policy makers to support HCP during pandemic response. |
|  | 3b. What organizational policies (new or revised) are needed to support HCP (including ancillary/support personnel) during the COVID-19 pandemic? How do organizational policies (e.g. time-off policies, sick time, attendance bonuses) affect HCP presenteeism? | HCPs are exposed to COVID-19 in and out of the workplace. Becoming ill with COVID-19 may lead to a HCP being out of work for extended periods of time, thus sick time, bonuses may be needed to encourage people to not work while ill. | Survey of HCPs and hospital administrators understanding the types of policies present to support HCPs. |  |
|  | 3c. How do organizational policies (e.g. time-off policies, sick time, attendance bonuses) affect HCP presenteeism? How do polices in place, but implicitly not supported by peers or leadership impact HCP mental health? | An understanding of the impact of policies in actual presenteeism rates are needed to understand their effectiveness.  For example, sick time policy in place but peer pressure/normative culture is presenteeism. HCP who call in sick experience guilt, stress, and internal conflict that can lead to moral injury and undermine resilience. | Survey, before/after observational studies of HCP working while ill rates (before/after sick time, COVID-19 pay, etc.).  Qualitative/ethnographic methods to assess causes and underlying culture of presenteeism in an organization.  Suggested outcome measures: Rates of HCPs working while ill before/after workplace policies are installed.  Themes for presenteeism, barriers and facilitators for use of time-off, sick time policies and unintended effects of attendance bonuses.^14^ | Enhanced policy and practice development for organizations to prevent presenteeism and support HCP mental health.^14^ |
| **RETURN TO WORK** | | | | |
| **1. Determine risk of SARS-CoV-2 transmission by returning HCPs to coworkers and patients, by healthcare worker type and setting** | What are the transmission risks associated with HCP returning to work and how does this vary by worker type, setting, and patient population? | Risk of COVID-19 transmission from HCP returning to work varies according to type of work and setting in which it is being done and the patient population the HCP works with. For example, the risk of transmission by a surgeon compared to a nurse or an EVS worker is likely different. Similarly, the risk of a nurse returning to work in the Oncology unit is likely higher than a nurse who works in the rehab unit. Coworker interactions and culture also vary by worker type – for example, nurses and other HCP within a shift may eat together in close proximity whereas case managers or floating service providers may have less coworker interaction. | Prospective cohort studies to evaluate risk of recovering HCP returning to work and transmitting COVID-19 to patients and coworkers, with subgroup evaluation by HCP role, patient population served, unit/location where HCP work.  Clinical cohort studies evaluating viability of virus in HCP with a range of symptoms from mild to severe and underlying HCP risk factors (immunosuppression) that may impact when and which HCP can return to work and in what settings. | Incidence/Rates of transmission to coworkers or patients in various HCP types, units, settings upon return to work. |
| **2. Determine the optimal criteria and modifications necessary for earliest and safe return to work** | What criteria should be used to determine when a HCP is safe to return to work and what if any modifications are needed to minimize transmission risk upon worker return? | Though CDC has clearly outlined criteria for return to work, many hospitals and health systems are using various criteria with a mixture of test and symptom based strategies. Data are needed to clearly identify optimal criteria that can allow workers to safely return at the earliest opportunity. These criteria would take into account worker type, healthcare setting, and patient population served.  Some HCP provide highly specialized service to patients that cannot be replaced. Identifying above criteria will help inform strategies to return such workers at the earliest moment. | Prospective or retrospective cohort studies to determine transmission in those returning to work after test-based vs symptom based return in various worker types/settings. | COVID-19 incidence in workplace upon repatriation of workers.  Optimal criteria for return to work by worker role or setting. |
| **3. Determine the sociocultural impact of return-to-work and strategies for successful repatriation of workers.** | What are the sociocultural aspects of returning to work after COVID-19? What social stigmas do workers face? How can employers assure a non-discriminatory, accepting workplace environment? | Persons diagnosed with COVID-19 face many social stigmas including fear from others that a worker with COVID-19 is contagious even after they have recovered. This may impact the way returning HCP are treated and the type and level of engagement employers and coworkers may have with them. This in turn may impact a worker’s performance and advancement, possibly leading to burnout, turnover, or psychological impacts. | Qualitative methods including interviews and surveys of healthcare workers in different settings to assess stigma, stressors, wellness, and needs to enable successful return to work. | Description of sociocultural experiences of returning and impact on workplace behaviors, productivity, and advancement.  Prevalence and duration of social stigma associated with COVID-19 in the workplace.  Strategies to mitigate negative consequences, and improve worker safety, health, wellness and work-life integration during the COVID-19 pandemic. |

Abbreviations: COVID-19, coronavirus disease 2019; HCP, healthcare personnel; SARS-CoV-2, severe acute respiratory virus 2; SCI/D, spinal cord injury/disorder; VA, Veterans Affairs.

**Supplemental Table 5: Research Priorities in Pediatric Populations**

| **PRIORITY AREAS** | **RESEARCH QUESTION**  **(overarching)** | **CLINICAL RATIONALE** | **SUGGESTED METHODOLOGY/ POPULATION** | **INTENDED OUTCOMES** |
| --- | --- | --- | --- | --- |
| **Epidemiology of the disease** | What is the role of symptomatic and asymptomatic children in disease transmission? | SARS-CoV-2 is characterized by lower rates of infection and milder illness in children, which is different than many other respiratory viruses (e.g., influenza).  It is important to understand age-based differences in virus transmissibility and the role of asymptomatic transmission in children.  These data are important both for policies surrounding isolation precautions in healthcare settings, as well as critical to safely planning K-12 school re-opening. | Mathematical modeling studies evaluating variable impacts of children on transmission.  Observational studies with longitudinal serial serologic and RT-PCR testing across healthcare and community settings. | Prevalence of asymptomatic infection and potential for transmissibility across settings.    Estimate and mitigate transmission risk in healthcare settings and schools. |
| **Surveillance and Outcomes** | What are the long-term sequelae of symptomatic/asymptomatic acute SARS-CoV-2 infection in children? What are the risk factors for and long-term sequelae of MIS-C? | Children account for a small proportion of diagnosed COVID-19 cases, but a high prevalence of asymptomatic or mildly symptomatic infection.  Children appear to be uniquely, though not exclusively, impacted by Multisystem Inflammatory Syndrome (MIS).  Long term sequelae across the spectrum of SARS-CoV-2 clinical syndromes in children are unknown. | Population-based cross-sectional or longitudinal observational studies measuring seropositivity and PCR positivity, history of COVID-19 infection, and clinical and demographic characteristics.    Prospective cohort studies to compare long term sequelae among patients with MIS-C and symptomatic vs asymptomatic acute COVID-19.  Case control studies to evaluate risk factors for MIS-C among an at-risk cohort. | Risk factors for MIS-C  Long-term sequelae of MIS-C and acute COVID-19. |
| **Isolation practices** | 1) How effective are current symptom-based screening measures in predicting need for COVID-19 testing and isolation for:  a-inpatients?  b-outpatients?  c-pre-procedural?  2) Is symptom-based screening predictive of positive COVID-19 testing? | COVID-19 has a wide variety of clinical presentations in children, with many presenting with no symptoms or mild symptoms.    Assess the utility of clinical parameters in predicting COVID-19 test positivity including defining timeframes for developing symptoms in children. | Prospective observational studies in acute care hospitals and use of symptom diaries in outpatient settings.  Case control studies to compare symptoms in patients who tested positive vs. tested negative. | Sensitivity and specificity of individual signs and symptoms to warrant placement on isolation precautions and/or isolation units while awaiting results.  Development of a validated integrated metric/scale that integrates key signs/symptoms and has sensitivity and specificity sufficient for predicting infection that would aid in stratifying children needing isolation precautions and/or testing. |
| **Environmental disinfection** | Can viable SARS-CoV-2 virus be isolated from the stool of incontinent infants and children? If so, for how long after acute infection and to what extent are environmental surfaces contaminated? | The significance of fecal shedding on environmental contamination and disease transmission is unknown.  Understanding these factors will inform cleaning and disinfection practices in the clinical setting, as well as be broadly applicable to community settings (e.g., daycares). | Observational studies sampling stool and environmental surfaces with subsequent molecular and culture-based testing to establish presence and viability of virus. | Optimal environmental cleaning and disinfection approaches  Estimate the potential role for transmission based on fecal contamination. |
| **ASP** | How has the COVID-19 pandemic affected antibiotic prescribing in the pediatric outpatient setting? | Antibiotic prescribing patterns may be affected by changes in healthcare delivery (e.g., telemedicine versus in-person visits) as well as changes in the epidemiology of non-COVID-19 viral infections during the pandemic. | Observational studies characterizing the frequency and indications for antibiotic prescribing during the pandemic  Novel metrics for benchmarking antibiotic use prescribed by telemedicine robust to changes in case mix, absolute number of visits, and absolute numbers of prescriptions. | Identify conditions with excess variation in antibiotic use across centers to inform future targeted outpatient stewardship efforts.  Metrics for benchmarking antibiotic use prescribed through telemedicine. |

Abbreviations: COVID-19, coronavirus disease 2019; MIS-C, Multisystem Inflammatory Syndrome in Children; PCR, polymerase chain reaction; RT-PCR, reverse transcription polymerase chain reaction; SARS-CoV-2, severe acute respiratory virus 2.

**Supplemental Table 2: Research Priorities in Pediatric Populations**

| **PRIORITY AREAS** | **RESEARCH QUESTION**  **(overarching)** | **CLINICAL RATIONALE** | **SUGGESTED METHODOLOGY/ POPULATION** | **INTENDED OUTCOMES** |
| --- | --- | --- | --- | --- |
| **Epidemiology of the disease** | What is the role of symptomatic and asymptomatic children in disease transmission? | SARS-CoV-2 is characterized by lower rates of infection and milder illness in children, which is different than many other respiratory viruses (e.g., influenza).  It is important to understand age-based differences in virus transmissibility and the role of asymptomatic transmission in children.  These data are important both for policies surrounding isolation precautions in healthcare settings, as well as critical to safely planning K-12 school re-opening. | Mathematical modeling studies evaluating variable impacts of children on transmission.  Observational studies with longitudinal serial serologic and RT-PCR testing across healthcare and community settings. | Prevalence of asymptomatic infection and potential for transmissibility across settings.    Estimate and mitigate transmission risk in healthcare settings and schools. |
| **Surveillance and Outcomes** | What are the long-term sequelae of symptomatic/asymptomatic acute SARS-CoV-2 infection in children? What are the risk factors for and long-term sequelae of MIS-C? | Children account for a small proportion of diagnosed COVID-19 cases, but a high prevalence of asymptomatic or mildly symptomatic infection.  Children appear to be uniquely, though not exclusively, impacted by Multisystem Inflammatory Syndrome (MIS).  Long term sequelae across the spectrum of SARS-CoV-2 clinical syndromes in children are unknown. | Population-based cross-sectional or longitudinal observational studies measuring seropositivity and PCR positivity, history of COVID-19 infection, and clinical and demographic characteristics.    Prospective cohort studies to compare long term sequelae among patients with MIS-C and symptomatic vs asymptomatic acute COVID-19.  Case control studies to evaluate risk factors for MIS-C among an at-risk cohort. | Risk factors for MIS-C  Long-term sequelae of MIS-C and acute COVID-19. |
| **Isolation practices** | 1) How effective are current symptom-based screening measures in predicting need for COVID-19 testing and isolation for:  a-inpatients?  b-outpatients?  c-pre-procedural?  2) Is symptom-based screening predictive of positive COVID-19 testing? | COVID-19 has a wide variety of clinical presentations in children, with many presenting with no symptoms or mild symptoms.    Assess the utility of clinical parameters in predicting COVID-19 test positivity including defining timeframes for developing symptoms in children. | Prospective observational studies in acute care hospitals and use of symptom diaries in outpatient settings.  Case control studies to compare symptoms in patients who tested positive vs. tested negative. | Sensitivity and specificity of individual signs and symptoms to warrant placement on isolation precautions and/or isolation units while awaiting results.  Development of a validated integrated metric/scale that integrates key signs/symptoms and has sensitivity and specificity sufficient for predicting infection that would aid in stratifying children needing isolation precautions and/or testing. |
| **Environmental disinfection** | Can viable SARS-CoV-2 virus be isolated from the stool of incontinent infants and children? If so, for how long after acute infection and to what extent are environmental surfaces contaminated? | The significance of fecal shedding on environmental contamination and disease transmission is unknown.  Understanding these factors will inform cleaning and disinfection practices in the clinical setting, as well as be broadly applicable to community settings (e.g., daycares). | Observational studies sampling stool and environmental surfaces with subsequent molecular and culture-based testing to establish presence and viability of virus. | Optimal environmental cleaning and disinfection approaches  Estimate the potential role for transmission based on fecal contamination. |
| **ASP** | How has the COVID-19 pandemic affected antibiotic prescribing in the pediatric outpatient setting? | Antibiotic prescribing patterns may be affected by changes in healthcare delivery (e.g., telemedicine versus in-person visits) as well as changes in the epidemiology of non-COVID-19 viral infections during the pandemic. | Observational studies characterizing the frequency and indications for antibiotic prescribing during the pandemic  Novel metrics for benchmarking antibiotic use prescribed by telemedicine robust to changes in case mix, absolute number of visits, and absolute numbers of prescriptions. | Identify conditions with excess variation in antibiotic use across centers to inform future targeted outpatient stewardship efforts.  Metrics for benchmarking antibiotic use prescribed through telemedicine. |

Abbreviations: COVID-19, coronavirus disease 2019; MIS-C, Multisystem Inflammatory Syndrome in Children; PCR, polymerase chain reaction; RT-PCR, reverse transcription polymerase chain reaction; SARS-CoV-2, severe acute respiratory virus 2.

**1.** Shanafelt T, Ripp J, Trockel M. Understanding and Addressing Sources of Anxiety Among Health Care Professionals During the COVID-19 Pandemic. *JAMA* 2020;323:2133-2134.

**2.** Wu Y, Wang J, Luo C, et al. A Comparison of Burnout Frequency Among Oncology Physicians and Nurses Working on the Frontline and Usual Wards During the COVID-19 Epidemic in Wuhan, China. *J Pain Symptom Manage* 2020;60:e60-e65.

**3.** Turk MA, Landes SD, Formica MK, Goss KD. Intellectual and developmental disability and COVID-19 case-fatality trends: TriNetX analysis. *Disabil Health J* 2020;13:100942.

**4.** Costantini A, Mazzotti E. Italian validation of CoViD-19 Peritraumatic Distress Index and preliminary data in a sample of general population. *Riv Psichiatr* 2020;55:145-151.

**5.** Restauri N, Sheridan AD. Burnout and Posttraumatic Stress Disorder in the Coronavirus Disease 2019 (COVID-19) Pandemic: Intersection, Impact, and Interventions. *J Am Coll Radiol* 2020;17:921-926.

**6.** Borges LM, Barnes SM, Farnsworth JK, Bahraini NH, Brenner LA. A commentary on moral injury among health care providers during the COVID-19 pandemic. *Psychol Trauma* 2020;12:S138-S140.

**7.** Apisarnthanarak A, Apisarnthanarak P, Siripraparat C, Saengaram P, Leeprechanon N, Weber DJ. Impact of anxiety and fear for COVID-19 toward infection control practices among Thai healthcare workers. *Infect Control Hosp Epidemiol* 2020;41:1093-1094.

**8.** Civantos AM, Byrnes Y, Chang C, et al. Mental health among otolaryngology resident and attending physicians during the COVID-19 pandemic: National study. *Head Neck* 2020;42:1597-1609.

**9.** Hou T, Zhang T, Cai W, et al. Social support and mental health among health care workers during Coronavirus Disease 2019 outbreak: A moderated mediation model. *PLoS One* 2020;15:e0233831.

**10.** Lu W, Wang H, Lin Y, Li L. Psychological status of medical workforce during the COVID-19 pandemic: A cross-sectional study. *Psychiatry Res* 2020;288:112936.

**11.** Zerbini G, Ebigbo A, Reicherts P, Kunz M, Messman H. Psychosocial burden of healthcare professionals in times of COVID-19 - a survey conducted at the University Hospital Augsburg. *Ger Med Sci* 2020;18:Doc05.

**12.** Rauch SAM, Simon NM, Rothbaum BO. Rising tide: Responding to the mental health impact of the COVID-19 pandemic. *Depress Anxiety* 2020;37:505-509.

**13.** Fessell D, Cherniss C. Coronavirus Disease 2019 (COVID-19) and Beyond: Micropractices for Burnout Prevention and Emotional Wellness. *J Am Coll Radiol* 2020;17:746-748.

**14.** Shale S. Moral injury and the COVID-19 pandemic: reframing what it is, who it affects and how care leaders can manage it. *BMJ Leader* 2020:leader-2020-000295.
